# Improving recombinant protein productivity in CHO cells via multi-omics data integration

**DOI:** 10.1186/s40643-026-01123-3

**Published:** 2026-08-31

**Authors:** Yuan Shen, Lei Shi, Xi Zhang, Xiao Guo, Wei-hua Dong, Tian-yun Wang

**Affiliations:** 1 School of Pharmacy, Henan Medical University, Xinxiang, 453003 People’s Republic of China; 2 Henan Key Laboratory of Neurorestoratology and Protein Modification, Henan Medical University, Xinxiang, 453003 China; 3 School of Basic Medical Sciences, Henan Medical University, Xinxiang, 453003 People’s Republic of China; 4 Henan Higher Medical Education Development Research Center, Henan Medical University, Xinxiang, 453003 People’s Republic of China

**Keywords:** CHO cells, Bioprocessing, Omics, Cell engineering, Process optimization

## Abstract

Chinese hamster ovary (CHO) cells represent the dominant host system for the production of recombinant therapeutic proteins. In recent decades, extensive research has focused on process/media optimization and cell line engineering to improve both the productivity and quality of biopharmaceutical proteins produced in CHO cells. Nevertheless, the inherent complexity of biological pathways and the heterogeneous cellular responses to different environmental conditions have posed substantial challenges to traditional methodologies. Recent advances in omics technologies have enabled comprehensive characterization of CHO cell physiology, providing multidimensional molecular and phenotypic insights that facilitate the enhancement of recombinant protein production. This review first summarizes the methodologies and advances in CHO omics research, including genomics, transcriptomics, proteomics, metabolomics, and epigenomics. It then examines contemporary approaches to integrate and analyze multi-omics data in CHO cells. The review further elucidates how these multi-omics datasets can be strategically applied across various developmental stages, including cell line selection, genetic engineering, expression vector design, and bioprocess optimization. Finally, we explore the transformative potential of integrating multi-omics with artificial intelligence and discuss promising future research directions in CHO cell studies. These emerging paradigms offer novel opportunities for data-driven cell engineering and bioprocess optimization in CHO-based biomanufacturing.

**Graphical abstract:**

**Supplementary Information:**

The online version contains supplementary material available at https://doi.org/10.1186/s40643-026-01123-3.

**Figure:**
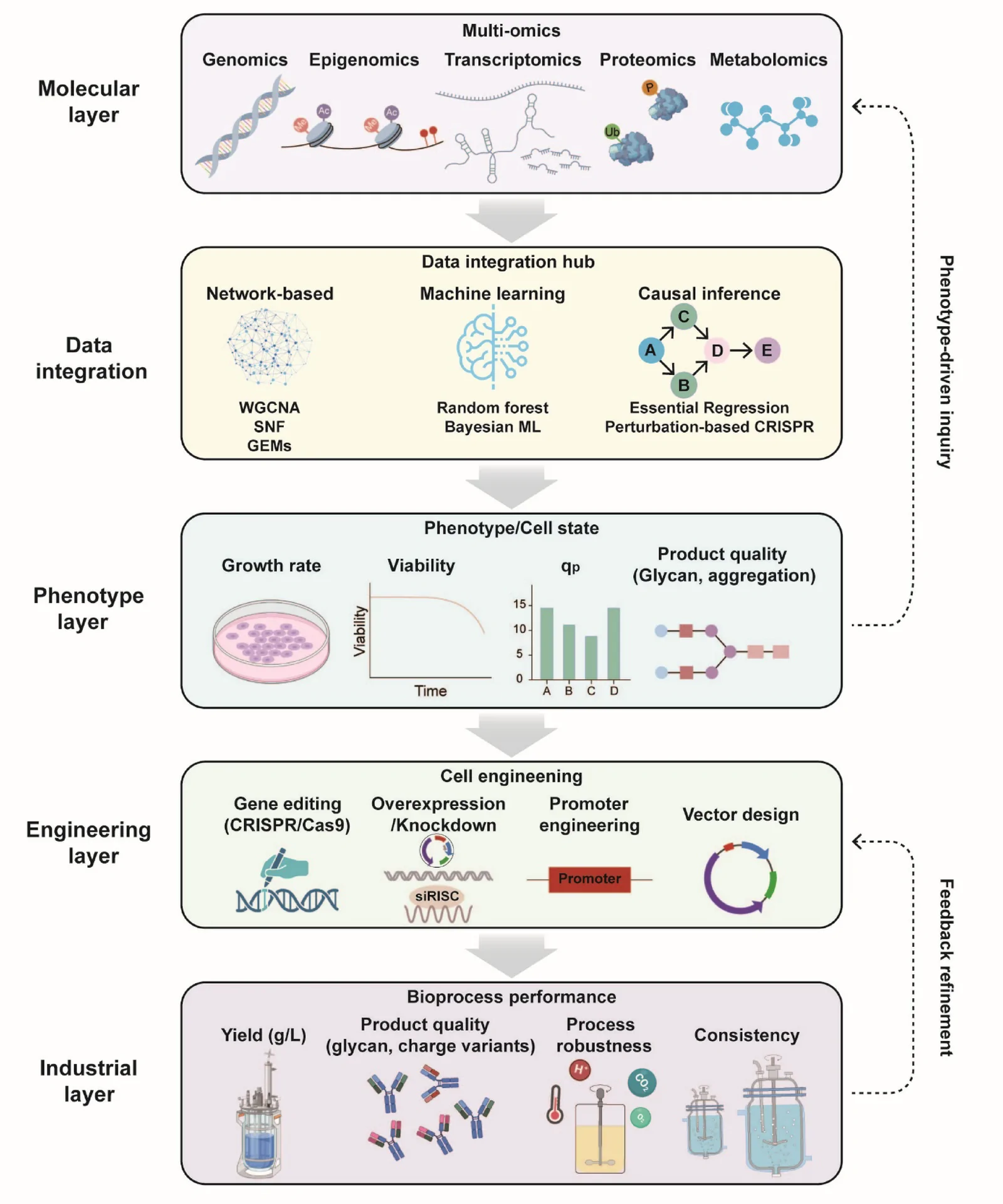

## Introduction

Recombinant therapeutic proteins (RTPs) have been extensively used to treat various diseases. Since the landmark approval of tissue plasminogen activator (tPA) in 1987, the first recombinant protein produced in mammalian cells, the biopharmaceutical market has experienced exponential growth (Kinch 2015). Nowadays, more than 400 biopharmaceutical products have received FDA approval (Walsh and Walsh 2022; de la Torre and Albericio 2024), with therapeutic antibodies dominating the market. As of 2021, therapeutic antibodies accounted for 15 of the 20 best-selling drugs worldwide, representing a global market value of $217 billion (Walsh and Walsh 2022).

Such RTPs are typically produced in mammalian cell lines, among which Chinese hamster ovary (CHO) cells are the most popular mammalian expression host. Currently, over 70% of approved RTPs are produced in CHO cells (Walsh and Walsh 2022), largely owing to several key features that make them well‑suited for industrial production. (i) Allow large-scale industrial bioprocess in suspension culture with serum-free media (Tihanyi and Nyitray 2020). (ii) Facilitate genetic modification easily and achieve very high yields of RTPs, with titers exceeding 10 g/L in fed‑batch cultures (Zeh et al. 2024; Mahe et al. 2022). (iii) Produce complex proteins with human-like post-translational modifications (PTMs). (iv) Enhance biosafety via lower susceptibility to human viral infection (Fischer et al. 2015). Given these attributes, CHO cells have become the dominant host for RTP production, driving extensive research into cell engineering strategies to further enhance titers and product quality.

The bioprocess of RTPs is complex and involves multiple steps, including transcription, translation, folding, assembly, transportation, and secretion. Although improvements in bioprocess performance have been documented, the underlying cellular machinery remains poorly characterized. Single‑omics approaches have provided valuable insights into CHO cell biology, but they inherently capture only a fraction of the regulatory complexity governing RTP production. Transcriptomics reveals gene expression changes without informing protein abundance or activity; proteomics measures final effectors but lacks regulatory context; metabolomics reflects phenotypic endpoints without revealing upstream drivers; and genomics provides the genetic blueprint but cannot predict dynamic cellular responses. Integrating multi-omics data is therefore essential to bridge these gaps. Multi-omics technologies can be applied to study CHO cells across multiple molecular layers, including genomics (integration sites, copy numbers), epigenomics (gene modifications), transcriptomics (mRNA expression), proteomics (protein abundance), and metabolomics (metabolite levels), each offering a distinct perspective on the intracellular dynamics that shape bioprocess‑relevant traits. Up to this point, researchers have generated a wealth of multi-omics data from CHO cells, gradually advancing our understanding of the molecular basis for high-performing production clones in bioprocess settings.

Over the past five years, several valuable reviews have summarized the progress and challenges in CHO cell genomics, transcriptomics, proteomics, and metabolomics (Singh et al. 2024; Jerabek et al. 2022a; Marx et al. 2022; Nguyen and Zimmer 2023; Coulet et al. 2022; Dahodwala and Sharfstein 2025; Grassi et al. 2025; Park et al. 2024). However, the majority of these works focused on individual omics technologies (Coulet et al. 2022; Singh et al. 2024; Marx et al. 2022; Jerabek et al. 2022a; Nguyen and Zimmer 2023; Park et al. 2024), technological advances in specific platforms (Dahodwala and Sharfstein 2025; Grassi et al. 2025), the use of omics in combination with CRISPR (Sorourian et al. 2024), or domain-specific applications such as cell engineering, media optimization, and bioprocess modeling (Orellana et al. 2021; Ranpura et al. 2025). A comprehensive review that systematically integrates multi-omics data and translates them into actionable strategies spanning the entire CHO biomanufacturing pipeline from cell line engineering to process optimization remains lacking. This review addresses this gap by emphasizing multi‑omics integration and outlining an AI‑enabled biomanufacturing paradigm, serving as a roadmap for translating multi‑omics data into actionable biomanufacturing knowledge.

To this end, we first summarize the methodologies and advances of multi-omics studies in CHO cells, covering genomics, epigenomics, transcriptomics, proteomics, and metabolomics. We then discuss the prevailing advantages and challenges in combining and interpreting multi-omics data. Furthermore, we demonstrate the application of these datasets in driving innovations across key CHO biomanufacturing domains, including cell line development, functional gene engineering, expression vector optimization, and process/media optimization. Finally, we outline future directions for enhancing RTP yield and quality through knowledge-driven biomanufacturing in CHO cells.

## Omics data of CHO cells

**Fig. 1:**
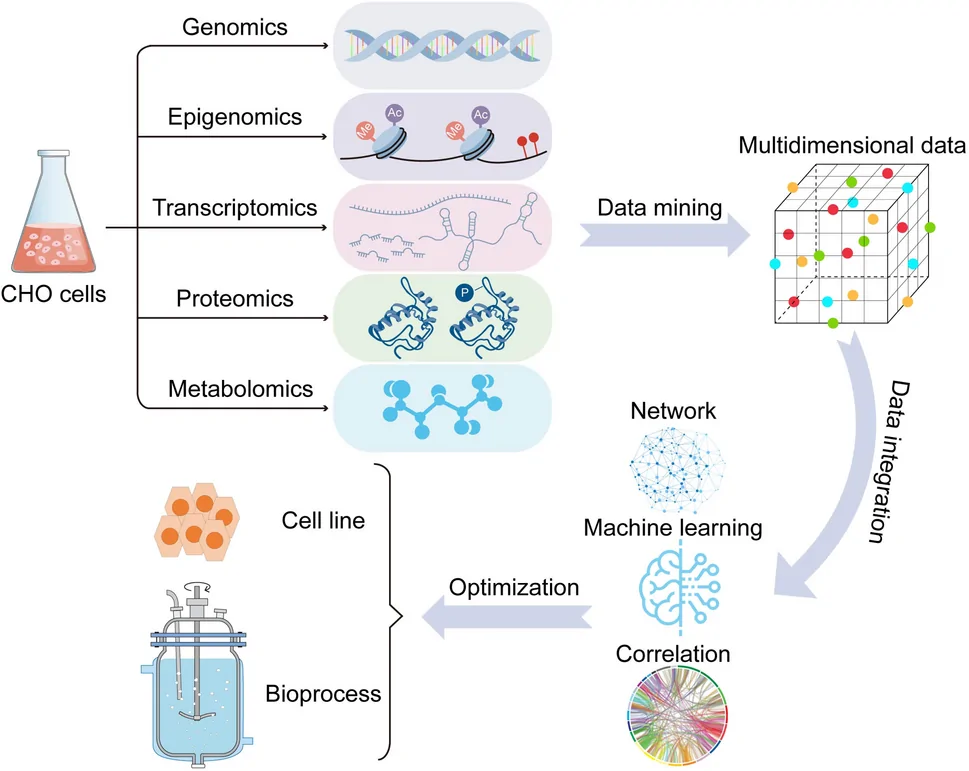
Schematic representation of multi-omics approaches in CHO cells towards the improvement in RTP productivity. Multi-omics technologies have generated large amounts of data in CHO cells. These multidimensional data can be analyzed in an integrative and interactive manner with the aid of machine learning. The advancement of multi-omics is expected to boost RTP productivity by facilitating future cell line engineering and bioprocess optimization

The rapid advancement of high-throughput sequencing has facilitated a surge in CHO multi-omics data. Systematic integration of these datasets will unlock rational strategies for bioprocess and cell line engineering, thereby boosting RTP production (Fig. 1). In the following sections, we highlight key omics-based methodologies and their applications in bioprocess development, and discuss recent progress toward multi-omics integration in CHO cell research.

### Genomic characterization

Genomic technologies enable comprehensive examination of genomic sequences and their variations, including single nucleotide variations (SNVs), insertion-deletions (indels), structural variations (SVs), and copy number alterations (CNAs). Industrial CHO cell lines, shaped by extensive mutagenesis and clonal diversification, display substantial genomic heterogeneity across these variation categories. Genomic characterization of various CHO cell lines provides a foundation for rational cell line development and engineering, enabling targeted integration of transgenes into safe harbor sites and supporting the identification of genomic features linked to high-performance phenotypes.

Prior to 2010, CHO genomic research was severely limited owing to the scarcity of reference sequence data. A landmark advance came in 2011 with the sequencing of the CHO-K1 cell line, which generated a 2.45 Gbp draft genome comprising approximately 24,000 annotated genes across 21 chromosomes (Xu et al. 2011). Subsequently, the genomes of diverse CHO cell lines were sequenced (Brinkrolf et al. 2013; Lewis et al. 2013; Kaas et al. 2015). In 2013, the genome of the Chinese hamster was sequenced and assembled, establishing a universal reference for all CHO cell lines (Brinkrolf et al. 2013). A refined version later emerged through a hybrid assembly approach incorporating material derived from Chinese hamster liver tissue and single-molecule real-time (SMRT) sequencing technology, yielding highly accurate scaffolds that closed 95% of gaps in the previous version (Rupp et al. 2018). A further leap forward occurred in 2020, when Hi-C-based chromosome conformation capture enabled the construction of chromosome-scale scaffolds, with 97% of the genome encompassed in 11 large scaffolds corresponding to Chinese hamster chromosomes (Hilliard et al. 2020). Currently, the CHO Reference Sequence (RefSeq) database is available at the National Center for Biotechnology Information (NCBI) for both Chinese hamster and CHO-K1 genomes, comprising 32,251 genes and 31,247 genes, respectively (https://www.ncbi.nlm.nih.gov/datasets/genome/GCF_003668045.3/; https://www.ncbi.nlm.nih.gov/datasets/genome/GCF_000223135.1/).

Despite remarkable technical advances, CHO genomics still faces significant challenges that constrain its translational impact. Current CHO genome assemblies remain incomplete, with repetitive regions poorly resolved and SVs systematically underdetected in short-read data. Genome architecture is highly host-line-specific, yet most studies focus on only one or two cell lines (primarily CHO-K1 and Chinese hamster, for which reference genomes are available), while other industrially relevant hosts such as CHO-DG44 and CHO-S remain understudied, limiting the generalizability of findings. Functional annotation relies heavily on orthology transfer from human and mouse, which may obscure lineage-specific adaptations. More critically, the link between specific genomic features and industrially relevant phenotypes such as productivity or growth remains poorly defined, and genomic variation is rarely analyzed in conjunction with transcriptomic or proteomic data to assess functional consequences. The dynamic nature of CHO genomes during long-term culture, including SVs and CNAs, is still largely unexplored, despite its direct implications for manufacturing stability. On the industrial front, while genomics has enabled practical outcomes such as the identification of safe harbor loci and genetic drift monitoring, whole-genome sequencing remains too costly for routine high-throughput screening, and regulatory frameworks lack clear guidelines for genomic data integration in cell line characterization. Addressing these gaps will require the establishment of standardized genomic variant compendia linked to bioprocess phenotypes, cost-effective targeted stability monitoring, and multi-omics integration to build predictive models of clone performance and stability.

### Transcriptomic data

Transcriptomic data refers to the profiles of RNA species, including mRNA, rRNA, tRNA, long-noncoding RNA (lncRNA), microRNA (miRNA), and others, generated from a given cell population at a given time. The primary objectives of transcriptomic studies in CHO cells are to quantify changes in transcript levels across different cell lines and culture conditions. The transcriptomic approaches mainly include hybridization-based microarrays and sequencing-based RNA-seq. Microarrays require prior knowledge of mRNA sequences and can only detect previously known genes and exons. Early CHO microarray studies relied on mouse or rat‑derived arrays due to the lack of a CHO genome sequence (Birzele et al. 2010), and a CHO-specific microarray platform was not reported until 2017 (Shridhar et al. 2017). However, microarrays could not detect low-abundance transcripts, identify novel transcripts, or distinguish highly homologous gene sequences. By contrast, RNA-seq offers low noise, broader dynamic range, and superior specificity, enabling detection of novel sequences, single-base variants, and low-abundance transcripts. With declining sequencing costs and advances in bioinformatics, RNA-seq has become the dominant approach in the CHO community (Chen et al. 2015). Nevertheless, both platforms face CHO‑specific interpretive challenges. RNA‑seq library preparation biases (e.g., GC bias and PCR duplicates) can obscure detection of low‑abundance transcripts, particularly those encoding transcription factors and metabolic enzymes, while normalization strategies may differentially impact outcomes across CHO host lines with markedly different karyotypes and expression profiles. Moreover, reproducibility across independent runs is especially concerning in CHO bioprocess studies, as batch effects arising from minor variations in cell banking, media lots, or bioreactor scales can overshadow subtle but meaningful transcriptional responses to process perturbations. Standardized spike‑in controls and rigorous quality metrics are increasingly recommended, but their routine implementation in CHO research is hindered by the absence of a universally accepted CHO reference genome and the limited availability of line‑specific quality control reagents.

In contrast to the static genome, the transcriptome is highly dynamic, differing across cell types, culture stages, and environmental conditions. Comparative transcriptomics has been widely applied to assess the impact of CHO cells under varied growth conditions, including temperature, pH, dissolved oxygen, and hydrodynamic stress, as well as culture media modifications, such as small molecular additives, media components and supplements, altered feeding patterns, and reduced toxic byproducts (Sieck et al. 2014; Shridhar et al. 2017; Gowtham et al. 2017; Chen et al. 2015; Kumar et al. 2021; Torres et al. 2023; Capella Roca et al. 2020). Transcriptomic analysis has been used to link gene expression changes in recombinant CHO lines to bioprocessing performance (Harreither et al. 2015; Raab et al. 2024), trace cell lineage evolution and identify biomarkers for phenotypic traits such as stable expression, growth rate, and productivity (Jamnikar et al. 2015; Clarke et al. 2011; Ogata et al. 2021; Desmurget et al. 2024). Furthermore, transcriptomic analysis has enabled reconstruction of key pathways in CHO cells, such as protein secretion, degradation, lipid metabolism, and carbohydrate metabolism, thereby supporting targeted engineering (Lund et al. 2017; Thalen et al. 2024; Rives et al. 2024). A recent multi-omics analysis of 95 CHO cultures expressing diverse RTPs confirmed that low-producing cells upregulate protein degradation pathways, whereas high-producing cells enhance lipid metabolism and oxidative stress responses (Masson et al. 2025).

Non-coding RNAs have attracted increasing interest for CHO cell engineering. miRNA transcriptome profiling has been performed under various conditions (Hackl et al. 2011; Hernandez Bort et al. 2012; Liu et al. 2022a), and several miRNAs have been reported to influence bioprocessing efficiency and RTP output in CHO cells, such as miR17, miR19b, miR23b, miR92a, miR30, miR3, miR143, miR200a, miR466h, miR483, miR557, miR574-3p, miR744, miR1287, and miR286 (Svab et al. 2021; Liu et al. 2022a). lncRNA landscapes have also been characterized, revealing productivity‑ and growth‑related lncRNAs such as Adapt15, GAS5, and PVT1 (Vito and Smales 2018; Vito et al. 2020; Motheramgari et al. 2020). Integrative analysis of lncRNA and mRNA transcriptomes has further identified candidate lncRNAs as selection markers or cell engineering targets (Novak et al. 2023). Despite these advances, functional validation of non‑coding RNAs remains challenging. Most studies are correlative, and the causal roles of specific lncRNAs or miRNAs in modulating productivity have not been systematically established. Furthermore, the specificity of miRNA mimics/inhibitors and lncRNA knockdown strategies is often poorly controlled, and off‑target effects are rarely assessed. These uncertainties, combined with the lack of robust genetic manipulation platforms suitable for industrial-scale applications, have limited the translation of non‑coding RNA findings into routine cell line engineering practice.

Beyond coding and non-coding RNAs, mRNA modifications, particularly N6-methyladenosine (m6A), have emerged as an additional regulatory layer.m6A critically modulates mRNA splicing, stability, and degradation in mammalian cells (Roundtree et al. 2017). Using integrated MeRIP-seq and RNA-seq, our group recently demonstrated a strong correlation between m6A modification patterns and transcriptomic profiles in CHO cells with differential RTP expression, underscoring the functional significance of m6A in enhancing RTP output (Wang et al. 2025). Nevertheless, these findings remain largely correlative, and direct causal evidence linking specific m6A sites to RTP productivity is lacking. Beyond technical bottlenecks such as antibody specificity and RNA input demands, the functional roles of m6A in CHO cells remain poorly understood. In particular, it is unclear whether m6A drives or merely correlates with gene expression changes, and how it interacts with translational control. This complicates the interpretation of m6A‑mediated regulation in industrial contexts.

In summary, transcriptomics has become an indispensable tool for understanding CHO cell physiology, enabling pathway reconstruction, biomarker discovery, and engineering target identification. However, the path from transcriptomic data to industrial implementation remains obstructed by multiple interconnected challenges. First, transcriptomics captures mRNA abundance but does not report downstream regulatory events, limiting its ability to predict RTP titers. Second, data integration is complicated by the diversity of CHO host lines, culture media, and analytical pipelines, which produce datasets that are difficult to reconcile across studies. Third, transcriptomic signatures are often validated under small‑scale laboratory conditions but fail to translate to large‑scale bioreactors, where hydrodynamic gradients and feeding heterogeneity introduce additional layers of complexity. From an industrial standpoint, transcriptomics has already contributed to media optimization and process characterization, yet its routine use in clone selection remains constrained by cost, turnaround time, and the lack of validated predictive models. Ultimately, the greatest translational value of transcriptomics in CHO engineering lies not in generating more descriptive datasets, but in building predictive, mechanistically anchored models that link gene expression to bioprocess outcomes, which can be iteratively refined through the design‑build‑test‑learn cycle. Achieving this vision will require community‑wide efforts to establish standardized reporting guidelines, develop CHO line‑specific reference materials, and integrate transcriptomics with other omics layers to capture the full spectrum of regulatory events that determine RTP production. Only through such multifaceted approaches can transcriptomics fulfill its promise as a true driver of CHO cell line engineering and bioprocess optimization.

### Proteomic profiling

Proteomics enables the large-scale, high-throughput analysis of all proteins and peptides, offering functional insights that can complement transcriptomics, particularly given the nonlinear correlation between mRNA and protein levels due to post‑transcriptional and post‑translational regulation. Early CHO proteomic studies relied on gel-based methods, including two-dimensional gel electrophoresis (2-DE) and two-dimensional differential gel electrophoresis (2D-DIGE), for detecting differential protein expression and post-translational modifications. However, these approaches suffered from limited capacity, sensitivity, resolution, and reproducibility, promoting a widespread shift toward liquid chromatography-tandem mass spectrometry (LC-MS/MS), which offers superior efficiency and accuracy (Noor et al. 2021). Leveraging the publicly available CHO-K1 genome, the first extensive proteomic profiling of CHO-K1 cells identified over 6000 proteins (Baycin-Hizal et al. 2012). Subsequent studies have expanded this depth, with a comprehensive spectral library now encompassing more than 10,000 CHO-K1 proteins (ProteomeXchange Consortium PXD016047) (Sim et al. 2020), demonstrating that previously unreachable proteomic depths have been achieved (Sim et al. 2020).

Building on these resources, comparative proteomic analyses have been extensively applied to investigate protein expression differences across CHO host cell lines (e.g., CHO-K1, CHO-S, CHO-DG44, and CHO-DXB11), revealing line-specific variations in proteins involved in growth, metabolism, translation, protein processing, and glycosylation (Baycin-Hizal et al. 2012; Xu et al. 2017; Lakshmanan et al. 2019). Proteomic comparison between CHO cells and Chinese hamster tissues has further highlighted the enrichment of DNA replication and protein synthesis pathways in CHO cells, which may underpin their rapid proliferation and high RTP production capacity (Heffner et al. 2020). A substantial body of proteomic research has also focused on phenotypic differences related to growth and productivity in response to various intrinsic or extrinsic stimuli, including cell culture phases (Carlage et al. 2012; Wei et al. 2011; Bryan et al. 2021a), conditioned media (Albrecht et al. 2018), transfected cell lines (Kuystermans et al. 2010; Lee et al. 1996; Meleady et al. 2012; Costello et al. 2018), and environmental perturbations such as temperature, pH, and dissolved oxygen (Kumar et al. 2008; Kim et al. 2011a; Van Dyk et al. 2003; Kantardjieff et al. 2010). Through these investigations, essential growth-related signaling pathways and candidate engineering targets have been identified. More recently, extensive proteomic studies have been conducted with other omics to dissect the high-productivity phenotype (Strasser et al. 2021; Kumar et al. 2022; Ali et al. 2020; Chevallier et al. 2020; Lee et al. 2021), providing an in-depth understanding of the cellular mechanisms of RTP production.

To achieve deeper subcellular resolution, the sub-proteome approaches (surfaceome, phosphoproteome, spatial proteome, and secretome) have been developed in CHO cells (Jerabek et al. 2022a; Nguyen and Zimmer 2023). Surfaceome has identified hundreds of surface proteins, including collagens, laminins, thrombospondin, and fibronectin, which connect extracellular signals to intracellular responses and may serve as engineering targets (Klingler et al. 2021; Jerabek et al. 2022b). Phosphoproteomic studies have been performed across different culture conditions (Henry et al. 2017; Chang et al. 2022a), culture states (Kaushik et al. 2018), and cell lines (Dahodwala et al. 2019; Schelletter et al. 2019; Bryan et al. 2021b), providing insight into signal dynamics and identifying potential engineering targets in CHO cells. In parallel, spatial omics proteomics has been used to profile organelles and large cellular structures in mAb-producing CHO clones with distinct productivity phenotypes, offering a comprehensive view of the secretory pathway and its associated proteins (Perez-Rodriguez et al. 2021; Kretz et al. 2022). Secretome studies have further revealed that CHO cells secrete over 1000 proteins, with a substantial fraction present in extracellular vesicles (Kumar et al. 2015; Keysberg et al. 2021; Busch et al. 2022; Liu et al. 2019; Pilely et al. 2020). Collectively, these sub-proteome approaches represent powerful tools for gaining subcellular-level insights and uncovering future engineering targets for CHO cell line development.

Despite these advances, CHO proteomics faces substantial limitations that impede its translational impact. A key constraint is the inherent trade‑off of each analytical platform. Gel‑based methods lack throughput and quantitative accuracy, while LC‑MS/MS suffers from dynamic range compression, where high‑abundance proteins mask detection of low‑abundance regulatory proteins relevant to productivity. Integrating proteomic and transcriptomic data remains equally challenging, as weak mRNA‑protein correlation, differences in measurement scales, missing values, and the lack of CHO‑specific computational frameworks complicate cross‑omics analysis. Reproducibility is further compromised by batch effects from cell banking, media lots, and sample processing, as well as by the genetic and phenotypic heterogeneity of CHO host lines, yet systematic quality controls and standardized reporting guidelines, such as those analogous to MIAPE (Minimum Information About a Proteomics Experiment), are not routinely implemented, limiting dataset comparability and reusability. Finally, industrial translation is constrained by high costs, lengthy workflows, scale‑dependent confounding in bioreactors, and the need for extensive functional validation of candidate proteins. Addressing these challenges will require standardized sample handling and reporting, CHO line‑specific reference databases, and systems‑level integration with other omics to generate mechanistically grounded, industrially actionable insights.

### CHO metabolomics

Metabolomics concerns the comprehensive analysis of small-molecule biochemicals (typically < 1500 Da) that represent the downstream outputs of cellular metabolic processes. CHO metabolomics typically includes accurate quantification of intracellular metabolites from washed cells and extracellular metabolites from debris-free culture supernatants, both of which vary with cell phases and culture conditions (Zhang et al. 2013). Quantitative metabolomics techniques used in CHO cells mainly include nuclear magnetic resonance (NMR) spectroscopy, mass spectrometry (MS), and near-infrared spectroscopy (Chong et al. 2012; Singh et al. 2024).

Early metabolomic studies in CHO cells focused on characterizing nutrient consumption and metabolite accumulation during culture. For instance, real-time metabolome profiling across various culture stages, particularly the transition from exponential growth to stationary phase, has identified specific metabolites as cell status biomarkers (Ahn and Antoniewicz 2011; Sengupta et al. 2011; Karst et al. 2017; Selvarasu et al. 2012; Templeton et al. 2013; Dean and Reddy 2013). Beyond profiling dynamic metabolic changes, metabolomics has been frequently applied to examine the effects of media formulations and process conditions on the cell metabolome, guiding improvements in cultivation procedures and rational medium design (Mohmad-Saberi et al. 2013; Dietmair et al. 2012; Sellick et al. 2015; Mulukutla et al. 2017). Furthermore, metabolomics has facilitated gene discovery and metabolic engineering by identifying specific metabolic signaling pathways linked to productivity enhancement (Chong et al. 2010; Mulukutla et al. 2017; Alden et al. 2020).

Metabolites and intermediates in primary metabolic pathways, such as glycolysis and the tricarboxylic acid (TCA) cycle, are closely related to CHO cell growth and cell-specific productivity (*q*_p_) (Dietmair et al. 2012; Coulet et al. 2022). Glycolytic metabolites, including glucose, glucose-6-phosphate, pyruvate, phosphoenolpyruvate, and fructose 6-phosphate, have been reported to accumulate during the exponential phage, presumably to meet the heightened energy demands of cell division (Sengupta et al. 2011; Templeton et al. 2013; Buchsteiner et al. 2018; Zhang et al. 2021; Zhu et al. 2023; Naik et al. 2023). Conversely, the stationary phase is often associated with elevated TCA cycle intermediates, such as citric acid, isocitric acid, α-ketoglutarate, and glutamate, which have been linked to higher *q*_p_ in CHO cells (Dietmair et al. 2012; Zhu et al. 2023). Accordingly, supplementation of TCA cycle intermediates (*i.*e., aspartate, citrate, glutamate, succinate, malate, and fumarate) has been shown to improve *q*_p_ and overall yield in CHO bioprocesses (Saldanha et al. 2023; Yao et al. 2021). In addition, high-producing CHO cells showed increased intracellular levels of NADPH, FAD, and glutathione (Chong et al. 2012), likely reflecting the role of these metabolites in linking TCA activity to endoplasmic reticulum (ER) homeostasis (Gansemer et al. 2020), which is critical for RTP production.

Despite these advances, the translational impact of CHO metabolomics is constrained by several critical limitations. First, analytical platforms have divergent shortcomings. NMR is reproducible but insensitive; MS is sensitive but suffers from ion suppression, matrix effects, and structural ambiguity, and near-infrared spectroscopy enables rapid measurements but lacks specificity (Singh et al. 2024). Second, metabolite identification lags behind other omics, as comprehensive CHO-specific spectral libraries remain unavailable, leaving the majority of detected features unannotated and biologically uninterpretable. Third, pre-analytical variability poses a critical challenge. Metabolic profiles shift rapidly during sampling due to ongoing enzymatic activity, while quenching and extraction conditions can introduce artifacts. Moreover, metabolites cannot be amplified, rendering each sample unique and irreplaceable. Fourth, data reliability is further undermined by pervasive batch effects and the absence of standardized libraries and reporting guidelines, limiting cross-study comparability. Finally, industrial adoption is hindered by the transient nature of metabolomic snapshots, the technical immaturity of real‑time bioreactor monitoring, and the need for ^13^C-based flux analysis to distinguish enzymatic regulation from substrate availability. Nevertheless, a growing number of studies have begun to translate metabolomic insights into actionable media engineering. Addressing these challenges will require coordinated efforts to expand CHO-specific spectral libraries, standardize sample handling protocols, and integrate metabolomics with flux analysis and other omics through systems biology approaches.

### Epigenomic modulation

Epigenomics encompasses the spectrum of covalent modifications to DNA and histone proteins that regulate chromatin structure and gene expression. Although CHO epigenomics is still in its infancy compared to genomic and transcriptomic counterparts, it is increasingly recognized as essential for elucidating CHO cell biology, particularly for understanding transgene expression levels and stability. In mammals, well-characterized repressive marks include DNA methylation, histone deacetylation, H3K9me3, and H3K27me3, while active marks include H3K4me3 and H3K27ac. Early studies on transgenic CHO cell lines established that instability of RTP expression could arise from epigenetic silencing of transgenes. For instance, DNA methylation at the transgene promoters contributes to reduced *q*_p_ in CHO cells (Kim et al. 2011b; Osterlehner et al. 2011; Yang et al. 2010). The histone modification levels at transgene loci correlate with mRNA expression and productivity (Paredes et al. 2013; Veith et al. 2016).

The development of next-generation sequencing, combined with conventional biochemical techniques, has propelled epigenomic profiling in CHO cells. Genome-wide histone modifications can be mapped using ChIP-seq and CUT&Tag, while chromatin accessibility is assessed via ATAC-seq and DNase I-seq. DNA methylation is profiled through bisulfite sequencing or array-based technologies. Higher-order chromatin structure is assessed using methods such as Hi-C (Rivera and Ren 2013). Application of these technologies has generated substantial datasets that provide a comprehensive understanding of epigenome responses to CHO cell evolution and bioprocesses.

In 2015, the first single-base-resolution DNA methylation map of mAb-producing CHO DP-12 cells revealed global hypomethylation relative to other mammalian methylomes (such as embryonic stem cells, their derivatives, and primary cells), with CpG island hypermethylation and partially methylated domains correlating with gene expression (Wippermann et al. 2015). Subsequently, Feichtinger et al. examined global DNA methylation and histone modification profiles across different evolutionary pressures and proposed that DNA methylation is primarily associated with establishing cell phenotypes, while rapid adaptations to environmental changes are largely orchestrated through histone modification dynamics (Feichtinger et al. 2016). DNA methylation was later shown to affect the metabolism-related genes and energy metabolism in different CHO cell lines, thereby influencing productivity phenotypes (Dhiman et al. 2019). Histone methylation also changes dynamically during batch culture, suggesting that rapid responses, such as nutrient limitation, may be executed through histone modification dynamics (Hernandez et al. 2019). Repressive histone marks (H3K9me3, H3K27me3 and DNA methylation) are highly enriched in non-expressed coding genes, whereas active marks (H3K4me3 and H3K27ac) are enriched on expressed genes (Hernandez et al. 2019), highlighting the crucial role of epigenetic regulation in gene transcription.

To investigate the extent of clonal heterogeneity in CHO cells and its epigenetic underpinnings, multiple subclones derived from the same parental CHO DP-12 line were cultured under identical conditions and compared. Each subclone developed a unique DNA methylation profile, particularly at transcription regulatory regions, along with significantly different gene expression patterns (Weinguny et al. 2021), highlighting the impact of epigenetic regulation on transcriptome diversity. Intriguingly, DNA methylation patterns are primarily derived from host cell lines (Chang et al. 2022b), indicating that cell clone selection has occurred at the beginning of cell line establishment via epigenetic regulation. Nevertheless, even subclones derived from the same parent exhibit divergent phenotypic and epigenomic characteristics (Weinguny et al. 2021), suggesting that epigenetic modulation is a cryptic but critical factor in generating high-performance CHO cell lines.

Nevertheless, several critical barriers currently limit the industrial translation of CHO epigenomics. These include prohibitive experimental costs, the difficulty of deriving causal relationships from predominantly correlative data, the intricate crosstalk among multiple epigenetic modifications, and the substantial effort required for functional validation of candidate targets. To provide a systematic overview, the strengths, limitations, data outputs, and industrial applications of each omics platform discussed above are summarized and compared in Table 1.

**Table 1:** Overview of CHO omics technologies and their applications

Omics type	Strengths	Weaknesses	Data Output	Cost	Throughput	Industrial applications
Genomics	Define stable intrinsic genetic background of CHO cells Identify safe integration sites Enable precise genome editing for cell line engineering Distinguish clonal genetic heterogeneity and enables stability traceability	Static DNA only—no dynamic process information Extensive CHO-specific variant annotations still incomplete No direct correlation with protein titer or quality; High cost limits industrial high-throughput screening	Final assembled genome (FASTA) Aligned sequencing reads (BAM) Genetic variant catalog (SNPs, Indels, CNVs in VCF format)	High (WGS: $500–$2000/sample; targeted: substantially lower)	Low (WGS: weeks‑scale, limited multiplexing; targeted: days‑scale, high multiplexing)	Identify and validate safe integration sites to reduce long‑term expression decline Characterize cell clones and control quality for genomic integrity and clonal traceability Discover genome‑wide targets for cell line engineering
Transcriptomics	Compare transcripts directly between high- and low-producing clones Profile regulation of key pathways (e.g., ER stress, apoptosis, secretion) Discover biomarkers for phenotypic traits (productivity, growth, stability) Elucidate cellular responses to culture conditions and perturbations	mRNA levels poorly predict protein abundance due to post-transcriptional regulation Transient expression fluctuations hinder stable biomarker identification Batch-to-batch variability confounds data interpretation	FASTQ raw reads and gene count matrix Differential transcripts and splice variants Pathway and functional enrichment tables	Moderate (RNA‑seq: $150–$500/sample)	Medium‑High (RNA‑seq: days‑to‑1‑week, 96‑well compatible)	Optimize media and processes based on dynamic transcriptomic signatures Accelerate clone screening via transcriptomic profiling Dissect mechanisms for targeted pathway engineering Identify biomarkers for robust cell line engineering
Proteomics	Provide the closest molecular readout to cellular function and phenotype Capture PTMs (glycosylation, phosphorylation) invisible to transcriptomics Directly quantify recombinant therapeutic proteins (RTPs) and host cell proteins (HCPs) Reveal rate‑limiting proteins in the key pathways for targeted engineering	Poor detection of low‑abundance proteins due to wide dynamic range MS inter‑batch variability and complex calibration High sample lability requiring stringent protocols Lower throughput and higher cost than transcriptomics	Raw MS spectra and protein / peptide abundance matrix PTM sites (phosphorylation, glycosylation, etc.) RTP glycosylation profiles (glycoform distribution)	High (MS‑based: $400–$2,000 / sample; targeted: lower)	Low–Medium (MS‑based: days‑to‑weeks; targeted: faster)	Quantify HCP and RTP abundance Characterize PTMs critical to RTP quality and HCP function Identify pathway bottleneck proteins for targeted engineering Establish protein‑level biomarkers for clone selection and process monitoring
Metabolomics	Monitor nutrient consumption and toxic byproduct (lactate, ammonia) accumulation in real‑time Correlate metabolic profiles with cell viability, q_p_, and titer Identify nutritional bottlenecks for media optimization Phenotype high‑producing clones for early selection	Poor traceability to upstream genetic causes Sampling artifacts due to short metabolite half‑lives Poor separation of intra‑ and extracellular metabolite pools Incomplete metabolome coverage and limited database annotations	Raw LC‑MS chromatograms and peak tables Metabolite quantification tables Kinetic profiles and pathway enrichment	Low–Medium (untargeted: $150–$400/sample; targeted: substantially lower)	Medium–High (LC‑MS: days‑to‑1‑week, autosampler‑ compatible for continuous runs; targeted: hours‑to‑days)	Develop serum‑free media and feed formulations Reduce toxic byproducts (lactate, ammonia) Optimize fed‑batch via real‑time metabolic profiling Metabolic phenotype clones for early‑stage selection
Epigenomics	Elucidate transgene silencing via DNA methylation and histone marks Characterize chromatin remodeling in response to culture perturbations Profile DNA methylation, histone modifications, and chromatin accessibility Profile epigenetic heterogeneity to predict clonal stability	High sensitivity to cell state and culture conditions Complex crosstalk among multiple marks Bioinformatics workflows complex and not yet routine No direct link to titer or product quality for standalone prediction	Methylation matrix and differentially methylated regions Chromatin accessibility peaks Histone modification profiles Chromatin interaction maps	Medium–High (~$300–$1500/sample, depending on assay)	Low–Medium (ATAC‑seq: days; ChIP‑seq/ CUT&Tag: days–1 week; WGBS/Hi‑C: 1–2 weeks)	Identify epigenetic signatures associated with high‑producing clones Monitor methylation dynamics for cell bank stability assessment Profile epigenetic heterogeneity for early clone selection Screen epigenetic‑modulating compounds to enhance and stabilize expression

## Multi-omics data analysis and integration in CHO

Although single-omics studies have advanced our understanding of CHO cell biology, analyses that rely on a single omics layer are inherently correlative and insufficient for comprehensive, systematic interrogation of cellular complexity. Recent studies have successfully employed multi-omics approaches to characterize global molecular landscapes and identify candidate targets through integration across multiple biological layers. These results enable a more holistic, mechanistic delineation of CHO cells and support community efforts to enhance the performance of this manufacturing host for RTP productivity.

Early multi-omics integration in CHO cells relied on basic statistical approaches, including correlation analysis, weighted gene co-expression network analysis (WGCNA), and multivariate regression (e.g., least absolute shrinkage and selection operator, LASSO), that correlate individual data layers but are limited to linear associations and cannot capture cellular complexity. Dimensionality reduction techniques such as principal component analysis (PCA) and t‑distributed stochastic neighbor embedding (t-SNE) have also been used for visualization, though they oversimplify integrated data. Table 2 summarizes early and recent multi-omics integration studies in CHO cells by strategy. These limitations spurred the development of more sophisticated methods, which we discuss below across four areas, including network-based approaches, machine learning, causal inference, and computational tools and databases.

**Table 2:** Representative CHO multi-omics integration studies classified by integration strategy

Multi-omics	Key feature/application		References
*Multi-omics association (foundational correlation-based methods)*			
Transcriptome Proteome		Revealed limited conserved signatures between high/low productivity clones	Bai et al. (2025)
Proteome Fluxome		DCA treatment improved bioprocess performance with minimal proteomic changes	Buchsteiner et al. (2018)
Phosphoproteome Proteome		Identified differential phosphoproteins in high vs. low q_p_ cells as potential engineering targets	Bryan et al. (2021a)
Transcriptome Metabolome		Prolonged culture drives modifications to proliferation, transcription, and metabolism	Torres et al. (2023)
Transcriptome Proteome Surfaceome Secretome		Systematic multi-omics comparison discovered 392 overexpression and 209 knockdown targets for CHO cell engineering	Raab et al. (2024)
Transcriptome m^6^A Methylome		Identified 668 differentially m^6^A-methylated peaks positively correlated with RTP expression	Wang et al. (2025)
*Network-based approaches*			
Transcriptome proteome		Contextualized CHO-GEM construction using GIMME for CHO-K1, CHO-S, CHO-DG44	Hefzi et al. (2016); Yusufi et al. (2017); Fouladiha et al. (2021); Calmels et al. (2019)
Transcriptome exometabolome		Contextualized CHO-GEM construction using mCADRE for CHO-S, CHO-DG44	Gutierrez et al. (2020); Gopalakrishnan et al. (2023)
Transcriptome Proteome Metabolome		Revealed that phenotypic heterogeneity may stem from genetic variations in cell cycle regulation	Lakshmanan et al. (2019)
Metabolome Transcriptome		Uncovered metabolic reprogramming and nutrient uptake bottlenecks via GEMs	Gopalakrishnan et al. (2024)
Multi-omics data integration and visualization		CHOmics: web-based platform supporting network-based integration (e.g., SNF) for CHO multi-omics data	Lin et al. (2020)
*Machine learning frameworks*			
Transcriptome (892 cell lines, 11 formats)		Elastic net regression identified Cnpy3 as stress biomarker correlated with productivity (r²=0.94)	Papadaki et al. (2025)
Metabolome Transcriptome		Multivariate ML discriminated high- and low-producers; TCA intermediates as early-stage biomarkers, amino sugar pathways as late-stage discriminators	Barberi et al. (2022)
Transcriptome Proteome Metabolome		Bayesian ML identified multi-omic panel (4 genes, 8 proteins, 11 metabolites) distinguishing high-producing cultures	Gurazada (2024)

### Network-based approaches

Network-based approaches project integrated multi-omics data onto biological networks, such as protein-protein interaction (PPI) networks, co-expression networks, gene regulatory networks, metabolic networks, and signaling networks, for downstream analysis. In CHO research, metabolic networks have been particularly prominent, with a growing repertoire of genome-scale metabolic models (GEMs). The community‑consensus model iCHO1766, published in 2016, served as an initial backbone for contextualizing multi-omics data (Hefzi et al. 2016). This foundation was subsequently expanded through a series of updated generic models with increasing gene and reaction coverage, including iCHO2291, iCHO2048s, iCHO2101, and iCHO2441, along with contextualized versions for specific cell lines such as CHO-K1, CHO-DG44, and CHO-biomass models (Park et al. 2024). The most recent addition to this lineage is iCHO3K (11,004 reactions, 3597 genes), which further expands the metabolic and secretory pathway coverage to support mechanistic analysis of CHO cell metabolism (Di Giusto et al. 2026). To generate context‑specific metabolic models, multi‑omics data have been integrated into CHO‑GEMs using dedicated algorithms such as GIMME (gene inactivity moderated by metabolism and expression), iMAT (integrative metabolic analysis tool), mCADRE (metabolic context‑specificity assessed by deterministic reaction evaluation), and CORDA (cost optimization reaction dependency assessment). These methods leverage transcriptomic, proteomic, and/or exometabolomic data to define reaction activity states and constrain flux solutions, enabling the prediction of cell line‑specific metabolic capabilities and the identification of bottlenecks (Calmels et al. 2019; Fouladiha et al. 2021; Gopalakrishnan et al. 2023; Yusufi et al. 2017). Signaling networks are equally important. Transcriptomic and surfaceome profiling of CHO cells has revealed enrichment of transcripts and surface receptors associated with PI3K/AKT, MAPK, JAK‑STAT, and RAP1 signaling pathways, highlighting the molecular basis for the robust growth and survival phenotypes observed in bioprocess environments (Jerabek et al. 2022b). Complementing these findings, systematic integration of transcriptomic, proteomic, metabolomic, and fluxomic data has identified multiple apoptosis‑related gene targets in CHO cells, including Ddah1, Tgm2, Clu, Tpt1, and Lgals1, many of which function within these same signaling networks (Orellana et al. 2021). PPI networks have also been applied to guide stable expression locus screening. By integrating chromosomal location and expression data with Gene Ontology (GO)/Kyoto Encyclopedia of Genes and Genomes (KEGG) pathway analysis and betweenness centrality evaluation, 25 candidate loci were identified, three of which (LIG4, LOC100759874, AASS) maintained > 95% EGFP‑positive rates after 40 generations (Liu et al. 2026).

The analytical algorithms applied to these networks include propagation/diffusion methods, network similarity-based methods (e.g., similarity network fusion, SNF), and network inference (e.g., WGCNA) (Jiang et al. 2025). Among these, network inference and similarity-based approaches have been particularly productive in CHO research. In one early study, WGCNA, a representative network inference method, was applied to 295 CHO cell microarrays, identifying six co‑expression modules associated with growth rate and *q*_p_, with functional enrichment in cell cycle, protein secretion, and vesicle transport (Clarke et al. 2011). Subsequent studies have extended network-based integration beyond transcriptomics to include proteomic and metabolomic data. Integrated transcriptomic, proteomic, and metabolomic analysis revealed that phenotypic heterogeneity in CHO cell populations is largely driven by inherent genetic variations, particularly in genes regulating cell cycle homeostasis (Lakshmanan et al. 2019). In another example, metabolomics, transcriptomics, and genome‑scale metabolic network analysis were combined across three antibody‑producing CHO cell lines, uncovering metabolic reprogramming and nutrient uptake bottlenecks that were not evident from any single data type alone (Gopalakrishnan et al. 2024). Additionally, CHOmics has emerged as a web‑based platform that supports network‑based integration strategies such as SNF, enabling integrative analysis and interactive visualization of CHO multi‑omics data (Lin et al. 2020). Collectively, these examples illustrate the translational potential of network‑based approaches for CHO cell line engineering and bioprocess optimization.

### Machine learning frameworks

Beyond descriptive network analysis, machine learning (ML) frameworks have emerged as powerful tools for building predictive models from multi-omics data. These methods are particularly valuable when the goal is not merely to identify differences between conditions, but to predict phenotypic outcomes, such as *q*_p_, growth rate, or product quality attributes, from molecular measurements. In CHO research, ML applications span three complementary paradigms: classic algorithms for feature prioritization and classification, deep learning for time-series prediction, and hybrid modeling that integrates mechanistic knowledge with data-driven learning.

In CHO applications, random forest-based approaches have been used to prioritize genomic and transcriptomic features by their contribution to productivity prediction, effectively reducing the feature space from thousands of candidates to a manageable set of high-confidence targets. A large-scale transcriptomics analysis of 892 monoclonal CHO cell lines producing 11 different antibody formats employed elastic net regression, a ML method combining LASSO and ridge penalties, to identify gene expression signatures correlated with productivity, revealing Cnpy3 as a novel stress biomarker strongly negatively correlated with product titer across complex formats (Papadaki et al. 2025). Metabolomics data have also been integrated with multivariate ML to discriminate high- and low-producing CHO clones, identifying TCA cycle intermediates as early-stage productivity biomarkers and amino sugar pathways as late-stage discriminators (Barberi et al. 2022).

A particularly noteworthy development is the convergence of mechanistic modeling with ML. Hybrid modeling frameworks that integrate ordinary differential equation models with constraint‑based metabolic modeling and ML components have demonstrated accurate prediction of viable cell density, product titer, and key metabolite concentrations across diverse CHO fed‑batch cultures, achieving generalization that neither purely data‑driven nor purely mechanistic models can attain (Ranpura et al. 2025). Bayesian ML integrating transcriptomics, proteomics, and metabolomics data from mAb-producing CHO cells has been used to predict overall mAb production potential, identifying a multi-omic panel of 4 genes, 8 proteins, and 11 metabolites capable of distinguishing high-producing cultures (Gurazada 2024). Beyond prediction, Bayesian optimization has been applied to navigate the high-dimensional media formulation space with substantially fewer experiments than traditional design-of-experiments approaches (Ndahiro et al. 2025), while physics-encoded transfer learning has addressed data scarcity in bioreactor scale-up by transferring knowledge from laboratory-scale models to industrial-scale bioreactors (Li et al. 2026). Collectively, these examples demonstrate the versatility of ML frameworks in both knowledge discovery and bioprocess optimization.

### Causal inference approaches

A critical limitation of current CHO multi-omics studies is their predominant reliance on correlative analyses, which cannot distinguish causal drivers from compensatory responses or bystander effects, a distinction that is central to identifying actionable engineering targets. To move beyond correlation, several frameworks have been developed to infer directed regulatory relationships by integrating multi-omics data with prior biological knowledge or modeling latent structures. One example is Essential Regression, a latent-factor framework for inferring causal relationships from multi-omics data without distributional assumptions (Bing et al. 2022). While such frameworks have primarily been applied in other mammalian systems, they can be adapted to CHO datasets to predict causal paths from genomic variants to transcriptomic changes to metabolic outputs, thereby providing testable hypotheses for experimental validation.

More recently, perturbation-based multi-omics has enabled systematic causal mapping of gene function at scale. Genome-scale CRISPR knockout screening in CHO-K1 host and recombinant cell lines has generated comprehensive datasets identifying essential genes involved in cell fitness and functional target genes associated with therapeutic protein production (Shin et al. 2025). Unlike observational multi-omics, which is inherently correlational, perturbation-based approaches actively perturb genes and measure resulting molecular changes, providing a direct route to causal inference. While systematic application of CRISPR screening combined with multi‑omics readouts to CHO cells is still emerging, this approach holds particular promise for identifying engineering targets with higher confidence than correlative screening alone. Together with observational methods such as Essential Regression, perturbation‑based strategies provide complementary causal insights, though they are not included in Table 2, which is restricted to multi‑omics integration studies. Collectively, these causal strategies are essential for moving CHO cell engineering beyond descriptive characterization toward mechanism-based target identification.

### Computational tools and databases for CHO multi-omics

As high-throughput omics technologies have advanced in CHO, some open-source tools and resources have been specifically designed for processing CHO omics data (Table 2). For example, ‘CHOgenome’ serves as a repository for genome-scale information on CHO cells, which also provides access in a searchable format and visualization of genomes and mRNA expression in CHO cells online (https://chogenome.org/index.php) (Kremkow et al. 2015). ‘CHO epigenome’ database encompasses the landscape of epigenome and the visualization in CHO cells in response to dynamic batch culture conditions (https://cho-epigenome.boku.ac.at/index.html) (Feichtinger et al. 2016; Hernandez et al. 2019). ‘CHOmine’ platform functions as an integrated data warehouse for CHO cells to query and analyze data from different sources (https://chomine.boku.ac.at/chomine/begin.do) (Gerstl et al. 2017). ‘CHOmodel’ is a valuable web-based resource that supplies the released CHO cell metabolic reconstructions (https://chomine.boku.ac.at/chomodel/organism/CHO) (Hefzi et al. 2016). ‘CHOGlycoNET’ dataset supplies a detailed glycan biosynthesis pathway map for CHO cells, presenting the vast majority of protein glycosylation profiles in CHO-K1 and CHO-S cells (https://data.mendeley.com/datasets/pph9ksfvjd/1) (Kotidis et al. 2023). In addition, many different computational tools and websites were established to aid the interpretation and visualization of omics data, such as Database for Annotation, Visualization and Integrated Discovery (DAVID), Gene Set Enrichment Analysis (GSEA), and Ingenuity Pathway Analysis (IPA) for pathway and gene enrichment analyses; STRING and Cytoscape for interactions in cellular networks.

Given the physiological and computational heterogeneity of CHO multi-omics data, conventional methods struggle to extract biological insights (Bersanelli et al. 2016). The emergence of multi-omics integration strategies is enabling a comprehensive understanding of CHO cell systems. ‘CHOmics’ is an online platform for interactive CHO cell analysis. (http://www.chomics.org) (Lin et al. 2020). This platform enables exploration coupled with the consolidation of multi-omics CHO data, additionally giving customers the option to personalize their analysis and interactively and systematically visualize the results (Lin et al. 2020). Besides this CHO-related platform, other common open-source platforms are established to aid non-programming researchers in integrating and analyzing CHO multi-omics data, such as Hiplot (https://hiplot.com.cn/home/index.html), OmicsAnalyst (https://www.omicsanalyst.ca/docs/Resources.xhtml), OmicsNet 2.0 (https://www.omicsnet.ca/) (Table 3). OmicsAnalyst, for example, provides a suite of tools for statistical integration, network integration, and causal analysis, enabling users to perform comprehensive multi-omics analyses without requiring programming expertise. Together, these integration strategies and omics resources enable data-driven CHO research and bridge biological insights with engineering strategies for cell line and bioprocess optimization.

**Table 3:** Online platforms for CHO multi-omics

Name	Website/resource	Function	References
*CHO cell-specific*			
CHOgenome	http://chogenome.org/	Collection of CHO genomic information, accession in a searchable format, and visualization of genomes	Kremkow et al. (2015)
	https://chogenome.org/proteome_new.php	Host proteomic data generated from CHO cell lines	
CHO epigenome	https://cho-epigenome.boku.ac.at/	CHO epigenome landscape and visualization	Feichtinger et al. (2016); Hernandez et al. (2019)
CHOmine	https://chomine.boku.ac.at/chomine/begin.do	Omics data warehouse for CHO cells and flexible queries	Gerstl et al. 2017)
CHOmodel	https://chomine.boku.ac.at/chomodel/organism/CHO	Reconstruction of CHO cell metabolism	Hefzi et al. (2016)
CHO.sf.net	https://cho.sourceforge.net/	Reconstruction of CHO metabolic network	Selvarasu et al. (2012)
CHOGlycoNET	https://data.mendeley.com/datasets/pph9ksfvjd/1	Glycosylation reaction network for CHO cells	Kotidis et al. (2023)
CHOmics	http://www.chomics.org	Integrative multi-omics data analysis and visualization for CHO	Lin et al. (2020)
*General-purpose tools*			
OmicsAnalyst	https://www.omicsanalyst.ca/docs/Resources.xhtml	multi-omics integration of transcriptomics, proteomics, and metabolomics	Zhou et al. (2021)
OmicsNet 2.0	https://www.omicsnet.ca/	multi-omics integration of genomics, transcriptomics, proteomics, and metabolomics	Zhou et al. (2022)
Paintomics4	https://paintomics.uv.es/	multi-omics integration of transcriptomics, epigenomics, proteomics, and metabolomics	Liu et al. (2022b)
Hiplot	https://hiplot.com.cn/home/index.html	multi-omics integration of genomics, transcriptomics, proteomics	Li et al. (2022a)

## Multi-omics data application in CHO cells

**Fig. 2:**
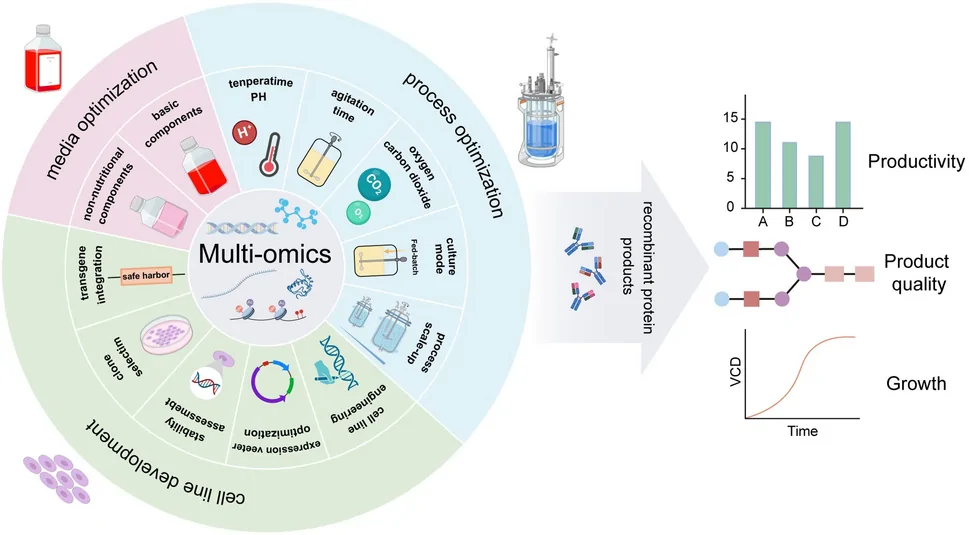
The application of CHO multi-omics in cell line construction, media formulation, and bioprocess configuration. The chart highlights several multi-omics applications relevant to CHO, covering areas in bioprocess and medium development, with respect to parameters such as media components, culture time, culture mode, and bioprocess scale. Multi-omics approach can also be applied to cell line establishment, encompassing host cell modification, expression vector optimization, cell stability assessment, clone selection, and transgene integration. The overarching aim of applying multi-omics within the CHO field is the achievement of improved cellular performance, high productivity, and enhanced product quality

With the aim of enhancing RTP productivity, multiple approaches have been established within the context of CHO bioproduction. These strategies primarily include cell line engineering, which modifies the physiological processes of producing cells at the gene level, and bioprocess engineering, which optimizes cell culture media and/or process conditions. The practical design and application of such strategies in CHO requires a comprehensive mechanistic understanding of the precise coordination and regulation of molecular events that drive bioprocess performance. The advancement of multi-omics offers a great opportunity to decipher these complicated molecular events by evaluating vast amounts of biological data. In this regard, it holds promise for enhancing the productivity of RTPs in CHO systems, benefiting both cell line engineering and bioprocess optimization (Fig. 2).

### Host cell line development and engineering

The development of high-producing CHO cell lines begins with the selection or construction of a suitable host cell background, followed by the stable genomic integration of the transgene. Multi-omics analyses have been instrumental in both stages by providing a systems-level understanding of host cell characteristics and guiding the identification of genomic loci that support robust and sustained transgene expression.

Since their initial isolation in 1957, CHO cells have given rise to various industrial sublines, including CHO-K1, CHO-S, CHO-DG44, CHO-DXB11, and CHO-GS, each of which exhibits distinct inherited characteristics and production potential. Multi-omics analyses have revealed substantial differences in metabolic flux, stress response, and secretory capacity among these sublines (Supplemental Table 1) (Lakshmanan et al. 2019; Xu et al. 2017; Raab et al. 2024; Heffner et al. 2020; Konitzer et al. 2015), providing a molecular basis for selecting appropriate hosts for specific production applications. Beyond subline selection, these datasets have traced the evolutionary trajectories of CHO lineages, identifying genetic and epigenetic alterations accumulated during domestication that correlate with desirable bioprocess phenotypes (Feichtinger et al. 2016; Tzani et al. 2021), thereby informing rational engineering of host cell lines.

In addition to host cell line selection, CHO cell line improvement involves generating stable CHO production cell lines through the genomic integration of exogenous DNA. The traditional method relies on random integration of transgene, which leads to high clonal variation and decreased clonal stability over production time (Zeh et al. 2024). Integration efficiency was significantly enhanced with the aid of the semi-targeted transgene integration systems, such as transposon systems (Sleeping Beauty, Leap-in, PiggyBac, Tol2) (Wei et al. 2022). However, transposon-assisted gene integration enables insertion into a region of interest but still lacks precise locus control. To achieve stable and high expression levels of RTPs, site-specific integration approaches, including zinc finger nucleases (ZFNs), transcription activator-like effector nucleases (TALENs), and the CRISPR/Cas9 system, have been developed to precisely insert transgenes into ‘safe harbors’, which are transcriptionally permissive and more stable than the bulk CHO genome (Hilliard and Lee 2023).

Currently, several genomic sites—including *Hprt1*, *Fer1L4*, *Hipp11*, *C12orf35*, *Mgat1*, and *GPIK1*, and a locus designated “genomic site A”—have been validated as safe harbors for gene integration in CHO cells (Zeh et al. 2024; Tihanyi and Nyitray 2020). Nevertheless, the identification and characterization of additional loci with potential for efficient, stable expression remain an active area of investigation. To this end, the transcriptomes and epigenomes have been used to systematically identify these novel safe harbor regions and analyze the stability characteristics in CHO cells, as summarized in Supplemental Table 1 (Hilliard and Lee 2021; Hertel et al. 2022; Pristovsek et al. 2019; Dhiman et al. 2020). Conventionally, RNA-seq and Targeted Locus Amplification (TLA) have been the primary tools for characterizing integration sites and expression profiles in CHO production cell lines (de Vree et al. 2014; Groot et al. 2021). More recently, long-read sequencing has complemented these approaches by directly deciphering intact transgenic loci and exposing the intricate structures of random integration events (Clappier et al. 2023). Together with transcriptomic and epigenomic analyses, these technological advances are now facilitating a more systematic evaluation of safe-harbor loci for stable RTP production.

### Functional gene engineering for enhanced bioprocess traits

While host cell line development focuses on establishing a stable genomic context for transgene expression, functional gene engineering targets specific cellular pathways to improve production-relevant phenotypes. Using multi-omics datasets, researchers have systematically identified and validated genes involved in key processes, such as apoptosis, metabolism, glycosylation, and protein secretion, which collectively determine the productivity and product quality of CHO cell lines.

Programmed cell death represents a major constraint on achieving high cell densities and prolonged culture durations in CHO bioprocesses. The molecular machinery of apoptosis has been extensively characterized through multi-omics studies, which have identified both pro- and anti-apoptotic regulators. Engineering CHO production hosts through overexpressing anti-apoptotic genes (such as *Bcl-2*, *XIAP*, *YAMA*,* HSP27*,* HSP70*,* HSPA5*,* Beclin-1*) or knocking-down/knocking-out proapoptotic genes (including *Bax*, *Bak*,* Caspase 3*, *Caspase 6*, *Caspase 7*, *AIF1*, *Alg-2*,* Requiem*) facilitate delaying apoptosis, extending culture viability, increasing integral viable cell density, and ultimately improving RTP production (Grilo and Mantalaris 2019; Zhang et al. 2025a, 2026). Beyond these well-characterized targets, integrative multi-omics analyses contribute to a comprehensive understanding of apoptosis-inducing responses and allow for the uncovering of novel candidate genes involved in apoptosis regulation, including *Psag*, *Tpt1*, *Tp53inp1*, *Rassf5*, *Ddah1*, *Tgm2*, *Clusterin*, *Anxa1*, *Lgals1*, *Hmxo1*, and *Psap*, as summarized in Supplemental Table 2 (Wong et al. 2006; Chong et al. 2011; Wei et al. 2011; Orellana et al. 2018, 2021; Carlage et al. 2012; Tossolini et al. 2018; Tzani et al. 2021). These discoveries have expanded the toolbox for apoptosis-based cell engineering, enabling more fine-tuned control of cell survival in bioprocess environments.

The metabolic activity of CHO cells directly influences cell growth and the accumulation of waste metabolites, particularly lactate and ammonia, which can inhibit cell proliferation and limit productivity. Multi-omics datasets, spanning metabolomics, fluxomics, transcriptomics and proteomics, have provided a comprehensive view of central carbon metabolism in CHO cells, revealing key regulatory nodes that can be manipulated to improve metabolic efficiency. Engineering strategies to reduce lactate accumulation include overexpressing pyruvate carboxylase (*PYC*) or downregulating key enzymes such as lactate dehydrogenase (*LDH*), pyruvate dehydrogenase kinase (*PDHK*), and malate dehydrogenase (*MDH*) (Li et al. 2022c; Zhang et al. 2026). In addition, omics-guided studies have also identified additional metabolic targets, including genes across diverse metabolic pathways, involved in fatty acid, glutathione, and cholesterol metabolism (Supplemental Table 2) (Blondeel et al. 2016; Selvarasu et al. 2012; Alden et al. 2020). This knowledge enables the rational manipulation of novel candidate genes to reprogram metabolic activity and enhance RTP production.

A substantial proportion of RTPs produced in CHO cells are glycoproteins, whose glycosylation profiles critically affect therapeutic efficacy, pharmacokinetics, and immunogenicity. Glycosylation engineering enables the generation of recombinant glycoproteins that better recapitulate human glycoforms (Yang et al. 2015; Wang et al. 2017; Heffner et al. 2021). Omics studies have provided deep insights into the intricate glycosylation machinery within CHO cells. Transcriptomic and glycomic analyses have revealed the expression patterns of glycosyltransferases and glycosidases across different culture conditions and cell lines, enabling the identification of pivotal genes governing glycan branching, sialylation, and fucosylation (Supplemental Table 2) (Sumit et al. 2019; Lee et al. 2021; Lakshmanan et al. 2019; Ali et al. 2020; Kotidis et al. 2023). The CHOGlycoNET dataset, a comprehensive glycan biosynthesis pathway map reconstructed from 200 glycan profiling datasets across multiple CHO cell lines, provides a valuable platform for investigating Golgi-localized glycosylation enzymes, accelerating glycomodel development, and predicting the outcomes of glycoengineering strategies (Kotidis et al. 2023). Collectively, these omics-informed efforts have enabled the engineering of CHO cell lines capable of producing glycoproteins with desirable characteristics.

In addition, the secretory capacity remains a bottleneck for the manufacturing of difficult-to-express RTPs in CHO cells. Overexpression of key secretory pathway proteins, such as SNAP-23, VAMP8, CERTS132A, XBP-1, HSPA5, PDI, GRP78, and ERGIC53, has been validated to enhance both the expression and secretion of RTPs in engineered CHO cell lines (Zhang et al. 2026). Omics studies have systematically mapped the transcriptional and proteomic responses associated with secretory stress, uncovering novel targets that regulate ER homeostasis, unfolded protein response (UPR), and vesicular trafficking (Lund et al. 2017; Raab et al. 2024; Kretz et al. 2022). These findings identify novel candidate targets (Supplemental Table 2) for engineering CHO cells with augmented secretory capacity, particularly for biotherapeutics that are intrinsically resistant to high-level production.

### Expression vector optimization

To enhance RTP yield in CHO cells, a commonly employed strategy lies in optimizing the expression vector’s transcriptional control elements, such as the promoter, enhancer, and polyadenylation (polyA) signal (Wang and Guo 2020; Romanova and Noll 2018; Li et al. 2018). Several reports have confirmed that various vector design approaches can influence the expression of the transgene (Yang et al. 2022b; Liu et al. 2024; Li et al. 2018; Zhang et al. 2024; Wang et al. 2022; Jia et al. 2019; Guo et al. 2020).

Although viral-derived promoters, such as human cytomegalovirus major immediate-early enhancer/promoter (hCMV) and simian virus 40 early promoter (SV40E), are widely used in CHO expression vectors, they are susceptible to epigenetic silencing and may trigger excessive stress responses. The identification of strong endogenous promoters through omics data has emerged as a compelling alternative. Integrative omics data from genomics, transcriptomics, and epigenomics have enabled the systematic discovery of strong endogenous promoters as listed in Supplemental Table 3 (Nguyen et al. 2019; Thaisuchat et al. 2011; Romanova and Noll 2018). For example, a temperature-sensitive promoter, S100a6, was identified through analysis of genes that were highly expressed in CHO transcriptome datasets (Thaisuchat et al. 2011). The activity of S100a6 promoter was conditionally triggered at low temperatures (basal productivity at 37 °C already higher than SV40E promoter), thus with the potential to enhance the production of hard-to-express proteins (Thaisuchat et al. 2011). Likewise, integrating RNA-seq with chromatin state data has revealed endogenous promoters with activities comparable to or exceeding those of viral promoters, enabling precise regulation of transgene expression (Nguyen et al. 2019). Besides viral and endogenous promoters, synthetic promoters present an intriguing strategy for application in CHO cells owing to the possibility of *de novo* engineering with customized abilities. The CHO multi-omics data have been utilized to guide the design of synthetic promoters (Cheng and Alper 2016; Johari et al. 2019; Brown et al. 2017). For instance, transcriptomic data have been used to identify transcription factor regulatory elements (TFREs) associated with highly expressed genes to rationally construct synthetic promoters, and the resulting promoters perform similarly to hCMV (Cheng and Alper 2016). In another study, CHO omics data were employed to identify novel TFREs that are potentially active at reduced temperature or in later culture stages, generating artificial promoters that outperformed the hCMV promoter during the biphasic production process (Johari et al. 2019).

Omics data have also been leveraged to identify active regulatory sequences naturally present at 5’ UTR and 3’ UTR regions of highly expressed endogenous genes, as shown in Supplemental Table 3 (Cao et al. 2021; Kirshina et al. 2023; Leppek et al. 2022; Liu et al. 2024). These sequences can be used to design and optimize UTRs in the vectors to boost RTP production. For example, incorporation of the *cis*-regulatory sequence iPLUS within the 3’ UTR of the recombinant vector doubled the trastuzumab production in ExpiCHO cells (Reis-Claro et al. 2024).

Engineering selection stringency on expression vectors is a convenient strategy for host cell line establishment. Augmenting RTP productivity in CHO cells is attainable through the attenuation of selective markers such as dihydrofolate reductase (DHFR) (Yang et al. 2022a). By analyzing miRNA transcriptomic data, let-7f is discovered to be highly expressed and invariant in CHO cells. Adding the let-7f target 3’UTR sequences to the DHFR gene on the expression vector can suppress DHFR expression and thus improve the product yield in CHO DHFR^−/−^ cell pools (Josse et al. 2018).

Moreover, the signal peptides greatly influence the efficient secretion of RTPs in CHO cells (Yang et al. 2023). The most suitable peptide sequence for RTPs can be designed by using omics data to identify crucial signal peptide elements from secreted native proteins (Supplemental Table 3). A recent study revealed that the utility of the designed signal peptide sequence in the vector can enhance productivity by more than 1.8-fold (O’Neill et al. 2023). Our research group further applied omics approaches to identify several novel signal peptides capable of enhancing human serum albumin (HSA) production in CHO cells (Zhang et al. 2025b). Beyond these vector component optimizations, multi-omics diagnostics can also address expression barriers at the sequence level. For example, an integrative workflow combining RNA sequencing, splicing prediction, codon optimization, and motif screening was applied to rescue expression of a difficult-to-express bispecific antibody. This approach identified aberrant splicing, ribosome pausing, and suboptimal codon usage as key liabilities; targeted sequence engineering subsequently yielded a variant with an 11-fold increase in stable titers (Gam et al. 2026). Collectively, these omics-informed vector design strategies have substantially improved the efficiency of transgene expression and host cell line establishment.

### Bioprocess optimization

Process engineering, alongside cell line engineering, represents another key approach for improving product yield and quality in CHO cell-based biomanufacturing (Lee et al. 2021; Li et al. 2022c). Multi-omics data can give mechanistic insights into cellular responses to environmental perturbations, enabling evidence-based optimization of culture media and process parameters (Stolfa et al. 2018; Farrell et al. 2014).

To satisfy demands of large-scale biopharmaceutical manufacturing, a variety of components are combined to formulate appropriate cell culture media that create the optimal physiological conditions for CHO cell growth (Combe and Sokolenko 2021; Li et al. 2022b; Lu et al. 2023). A number of omics studies, as summarzied in Supplemental Table 4, have been applied to elucidate the underlying mechanisms of CHO cell responses to various medium components, such as different glucose concentrations (Gowtham et al. 2017), cysteine concentrations (Ali et al. 2020; Greenfield et al. 2024), sodium butyrate treatment (Baik and Lee 2010; Muller et al. 2017; Schulze et al. 2022; Wippermann et al. 2017), hydrolysate supplementation (Kim et al. 2011a; Dhara et al. 2023; Kumar et al. 2021). These results contribute in-depth clarity on the functions of varying medium components, thereby guiding the appropriate media formulation to improve culture performance and production in CHO cells.

Environmental parameter optimization is also important for enhancing RTP yield and quality. The impact and mechanism of various bioprocess factors, such as pH (Lee et al. 2021), temperature (Henry et al. 2017; Baik et al. 2006; Fomina-Yadlin et al. 2015; Kumar et al. 2008), agitation (Sieck et al. 2014; Torres et al. 2021), culture time (Sumit et al. 2019; Hsu et al. 2017), scale-up (Gao et al. 2016; Torres et al. 2021), have been studied using omics approaches (Supplemental Table 4). These results enable the rational tuning of bioprocessing parameters to balance cell growth and protein production, as well as exploration of meaningful bioprocess marker genes for real-time process monitoring (Samoudi et al. 2021).

Fed-batch culture remains the predominant bioprocess operation mode for producing RTPs in CHO cells (Xu et al. 2023). Omics techniques have also been applied to monitor cellular physiological states throughout CHO cell fed-batch culture, identifying metabolic transitions, stress responses, and nutrient depletion signatures that correlate with declining productivity (Vodopivec et al. 2019; Selvarasu et al. 2012; Pan et al. 2019; Vito and Smales 2018; Alsayyari et al. 2018; Calmels et al. 2019). These results increase the awareness of fed-batch characteristics as well as critical process parameters that affect production, thus facilitating and expediting advances in CHO bioprocessing capacity (Supplemental Table 4). Beyond fed-batch, perfusion cultures, which enable continuous media exchange and prolonged culture durations, have benefited from omics-guided optimization of media exchange rates and nutrient feeding strategies (Agarwal et al. 2025). Collectively, multi-omics applied to bioprocess engineering has enabled the elucidation of intracellular pathways and responses to environmental perturbations, providing a systematic basis for the rational design of media and supplements, and culture process parameters for efficient CHO cell cultivation and high-yield biotherapeutics production.

## Conclusions and future perspectives

**Fig. 3:**
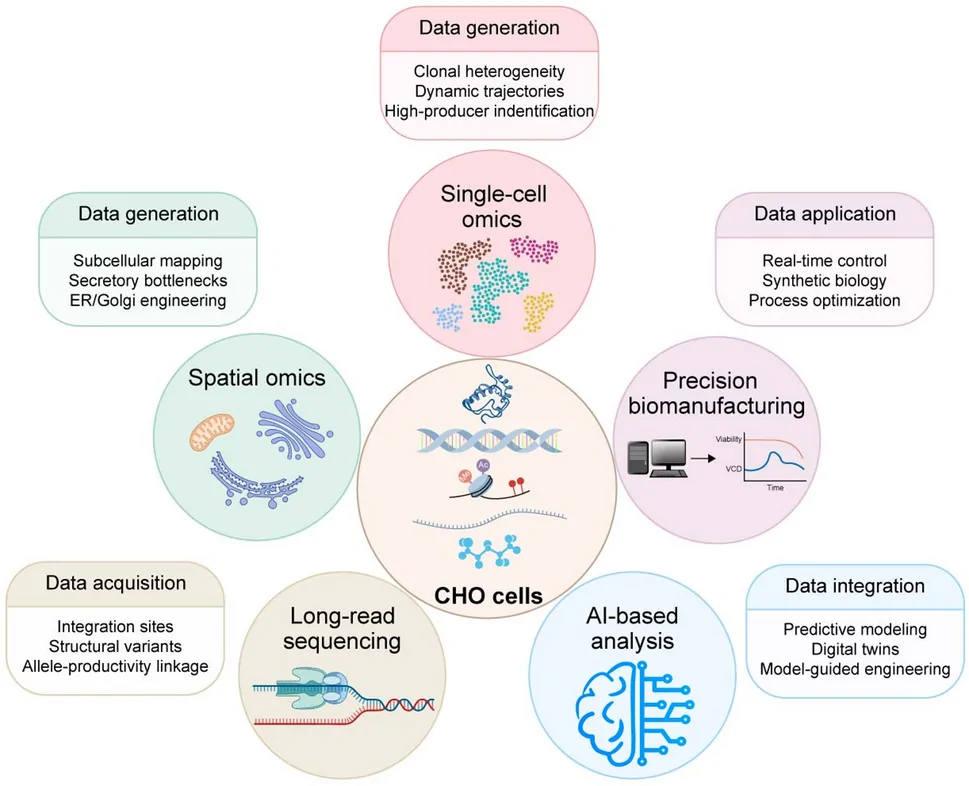
Future roadmap for multi‑omics‑driven CHO cell engineering. Single‑cell omics resolves clonal heterogeneity, reconstructs dynamic trajectories, and identifies high‑producer subpopulations. Spatial omics maps subcellular organization, reveals secretory bottlenecks, and enables ER/Golgi engineering. Long‑read sequencing resolves integration sites, detects structural variants, and links alleles to productivity traits. AI‑based analysis enables predictive modeling, digital twins, and model‑guided engineering. Precision biomanufacturing integrates real‑time control, synthetic biology, and process optimization to translate omics insights into industrial applications

CHO cells serve as the primary mammalian platform for producing RTPs and are expected to maintain their dominant position going forward. Multi-omics data have substantially expanded and strengthened the knowledge about the regulatory networks in CHO cell biology that drive further advancements in RTP production. In this review, we discuss omics studies in CHO cells, covering the progress of each single omics, the advances of multi-omics integration, and the application of multi-omics data in CHO engineering strategies. The CHO multi-omics data have elucidated the properties of high-producing cell lines and uncovered potential genetic intervention points for enhancing RTPs. Nevertheless, some challenges remain, which we have organized into five priority areas (Fig. 3).

### Single-cell multi-omics

Traditional omics analyses average signals across millions of cells, masking cellular heterogeneity that is clinically and biotechnologically relevant. In CHO cell lines, even those derived from a single clone, transcriptional and metabolic heterogeneity can give rise to subpopulations with divergent productivity, growth rates, and product quality. Single-cell omics technologies, particularly scRNA-seq, offer unprecedented resolution to dissect this heterogeneity and identify rare but high-producing subpopulations.

Compared with bulk RNA-seq, scRNA-seq enables deconvolution of cell-to-cell variability, reconstruction of dynamic trajectories (e.g., productivity decline or stress response during culture), and identification of marker genes specific to high-producing subpopulations for targeted engineering. Although still in its infancy in CHO research, scRNA-seq has already provided proof-of-concept insights, as demonstrated by observations that transcriptional drift contributes to clonal instability (Ogata et al. 2021), subclonal heterogeneity correlates with transgene variance and stress responses (Tzani et al. 2021), and culture conditions profoundly influence transcriptional homogeneity (Borsi et al. 2023).

Several challenges must be addressed before routine application. Cost remains prohibitive for large-volume bioreactor monitoring (~$1000–$2000/sample), though scRNA-seq is already feasible for small-scale clone screening during early cell line development. Technical parameters, including cell viability, sample processing, and dissociation, require optimization for CHO cells. Most critically, biological interpretation is greatly enhanced when scRNA-seq is paired with matched productivity and/or metabolomic data; without such integration, transcriptional heterogeneity cannot be definitively linked to functional phenotypes. Looking forward, the integration of scRNA-seq with CRISPR-based perturbation screens (Perturb-seq) represents a powerful frontier, enabling pooled genetic screening with single-cell readouts to systematically map gene function and identify engineering targets that drive cells toward desirable transcriptional states (Schraivogel et al. 2020). The emergence of single-cell multi-omics, simultaneously profiling transcriptome, epigenome, and proteome within the same cell, will further provide a comprehensive view of CHO cell heterogeneity and its functional consequences.

### Spatial omics

While single-cell omics resolves heterogeneity between cells, spatial omics technologies provide resolution within cells, capturing the subcellular localization of transcripts, proteins, and metabolites. In CHO cells, the secretory pathway, encompassing ER, Golgi apparatus, and vesicular trafficking machinery, is spatially organized, and its efficiency is directly tied to RTP production capacity. Subcellular omics techniques, including organelle-specific RNA-seq, proximity labeling proteomics, and spatial metabolomics, enable quantification of molecular entities within specific compartments such as the ER, mitochondria, and Golgi (Park et al. 2022; Eberwine et al. 2023). These spatially resolved data are critical for elucidating the mechanisms governing protein synthesis, folding, post-translational modification, trafficking, and secretion, all of which are inherently spatially constrained. Despite emerging investigations into the CHO cell secretome and surfacetome (Jerabek et al. 2022a), intracellular spatial omics analyses remain scarce (Kretz et al. 2022). Future efforts should prioritize mapping the subcellular distribution of transcripts and proteins in high- versus low-producing CHO clones, which could reveal compartment-specific bottlenecks that are invisible to bulk or even single-cell analyses. This knowledge would enable more precise engineering strategies, for example, enhancing ER chaperone capacity specifically in the ER lumen, or optimizing Golgi glycosylation enzymes without perturbing other compartments.

### Long-read sequencing

The predominance of short-read sequencing technologies (typically 75–400 bp) presents inherent limitations for resolving large-scale genomic alterations, repetitive regions, and structural variants in the highly rearranged CHO genome. Long-read sequencing platforms (e.g., PacBio and Oxford Nanopore), with reads averaging ~ 20 kbp, are increasingly being applied to CHO cell genomics, where they significantly enhance the detection of repeats, structural variations, and full-length transcript isoforms (Logsdon et al. 2020). For CHO research, long-read sequencing offers several transformative advantages. First, it enables the complete assembly of integration sites for transgenes, resolving complex multi-copy insertions that are common in random integration workflows and that influence clonal stability. Second, it facilitates the discovery of novel transcripts and splice variants that are missed by short-read sequencing due to mapping ambiguities in repetitive regions. Third, it provides unambiguous phasing of genetic variants, enabling the linkage of specific alleles to productivity traits. As long-read sequencing costs continue to decline, its integration into routine CHO cell line characterization and quality control is anticipated, particularly for assessing genomic stability across prolonged culture passages.

### Artificial intelligence and machine learning

The complexity of CHO multi-omics datasets increasingly demands computational approaches that go beyond traditional statistical methods to extract predictive and actionable insights. Artificial intelligence (AI) and ML are poised to transform CHO biomanufacturing across three interconnected axes: predictive modeling of cell phenotypes, AI-driven bioprocess digital twins, and AI-guided cell and media engineering.

For predictive modeling, ML algorithms have demonstrated utility in prioritizing features for productivity prediction from individual omics layers. For instance, transcriptomic analysis has identified stress biomarkers (Papadaki et al. 2025), while metabolomic profiling has revealed early- and late-stage discriminators of high-producing clones (Barberi et al. 2022). The next frontier lies in multi-omics ML models that integrate transcriptomic, proteomic, and metabolomic data to predict not only productivity but also product quality attributes such as glycosylation profiles and aggregation propensity. Bayesian approaches have provided proof of concept for such integration (Gurazada 2024). The key challenge now is translating these predictive models into practical screening tools for early-stage clone selection.

From static prediction, AI extends naturally into dynamic process optimization through digital twins, in silico representations that integrate multi-omics data with process parameters. GEMs provide a foundational mechanistic framework for such efforts, and hybrid modeling frameworks coupling GEMs with ML components have demonstrated robust generalization across process variations (Ranpura et al. 2025; Richelle et al. 2025). Physics-encoded transfer learning has emerged as a complementary strategy to address data scarcity in large-scale bioreactors (Li et al. 2026). Pilot implementations have shown feasibility in predicting key process metrics and optimizing media formulations, although fully integrated digital twins are not yet commercially available.

Beyond specific algorithms, AI is also reshaping the means by which we infer cellular objectives from omics data. The ObjFind-M framework infers reaction-level metabolic objectives directly from fluxomic and metabolomic data, revealing mitochondrial ATP synthase as a central metabolic driver in CHO cells (Morrissey et al. 2025). Bayesian optimization has enabled efficient navigation of high‑dimensional media formulation space with substantially fewer experiments than traditional approaches (Ndahiro et al. 2025). Collectively, these examples illustrate the dual role of AI in both extracting fundamental biological knowledge and enabling efficient experimental design, paving the way for fully autonomous CHO cell engineering platforms.

### Precision biomanufacturing and real-time process control

The convergence of multi-omics, AI, and automation is enabling a paradigm shift from retrospective analysis to real-time, predictive process control, a concept known as precision biomanufacturing. Advances in rapid analytical technologies, including Raman spectroscopy (Tanemura et al. 2023; Rubini et al. 2025) and automated flow cytometry (Corredor et al. 2025), now enable near‑real‑time monitoring of metabolites, viability, and product quality attributes. Integrating these data streams with ML models could enable dynamic process control, where feeding strategies, temperature shifts, or harvest times are adjusted in response to predicted cellular states rather than fixed schedules.

Synthetic biology, guided by multi‑omics insights, is expanding CHO cell engineering beyond traditional single‑gene strategies toward multiplexed, pathway‑informed engineering (Donaldson et al. 2022; Schulz et al. 2026) CRISPR‑Cas systems now enable multiplexed gene regulation and targeted integration of synthetic constructs into genomic safe harbors for predictable, stable transgene expression (Kalkan et al. 2023). GEMs provide a mechanistic scaffold for predicting metabolic outcomes of perturbations; the recent community‑consensus model iCHO3K exemplifies this resource (Di Giusto et al. 2026). Future efforts should couple GEMs with ML to create hybrid platforms that leverage both mechanistic knowledge and data‑driven learning. Despite these advances, barriers to industrial adoption persist, including high costs, specialized expertise, and lack of standardized protocols and data formats. Expanding community data‑sharing efforts with rich metadata, alongside closer academia–industry collaboration and user‑friendly software, will be essential to translate these technologies into routine biomanufacturing practice.

In summary, the future of CHO cell engineering lies in the convergence of single-cell and spatial omics, AI-driven integration, predictive modeling, precision biomanufacturing, and real-time process control. These technologies, individually and collectively, promise to transform CHO biomanufacturing from a largely empirical discipline into a predictive and precision-driven engineering endeavor. Given the remaining challenges, such as cost reduction, data standardization, model interpretability, sensor development, and industrial translation, the accelerating pace of technological innovation, together with growing community efforts to share data and tools, will be critical to realizing this vision in the coming decade. Success will require sustained collaboration between academia and industry, investment in infrastructure and training, and a commitment to open data and reproducible research practices.

## Supplementary Information

Below is the link to the electronic supplementary material.


Supplementary Material 1.


## Data Availability

Not applicable.
