# Supplementary material for: Improving recombinant protein productivity in CHO cells via multi-omics data integration
Source: Bioresour Bioprocess. 2026 Aug 31;13(1):124. doi: 10.1186/s40643-026-01123-3 (PMC13529640; doi:10.1186/s40643-026-01123-3)
Supplement: Supplementary file 1 [file 40643_2026_1123_MOESM1_ESM.docx]

**Supplemental Materials**

**Supplemental Table 1. Representative omics applications in host cell line development and engineering**

| **Development of host cell lines** | | | |
| --- | --- | --- | --- |
| Omics | CHO sublines | Key findings | Reference |
| transcriptome | 2 CHO-DG44 lines (BI-HEX-1 and BI-HEX-2) | The transcription of *Fut10*, *Gfpt2*, and *ST8Sia6* during antibody production in CHO-DG44 cells, along with constitutive *ST8Sia6* expression in BI-HEX-K1 cells | (Konitzer et al. 2015) |
| proteome | CHO-S  CHO-K1  CHO dhfr^-/-^ | Identification of key host cell proteins governing cellular growth kinetics and protein expression levels | (Xu et al. 2017) |
| transcriptome proteome  metabolome | CHO-K1  CHO-DXB11 CHO-DG44 | CHO cells exhibit intrinsic physiological variations, including cell-cycle deficits, reduced metabolism in DHFR-deficient lines (DG44/DXB11), and divergent protein processing and glycosylation capacities | (Lakshmanan et al. 2019) |
| proteome | CHO-S  CHO-K1  7 CH tissues | A comprehensive hamster proteomics atlas (8,470 proteins) reveals a substantial overlap in the proteomes of CHO-S and CHO DG44 lines, both enriched in cell cycle/nucleic acid processes | (Heffner et al. 2020) |
| transcriptom  proteome  miRNome  surfaceome  secretome | CHO cells  plasma cells | 392 potential overexpression targets and 209 candidates for knock-down or knock-out in CHO engineering | (Raab et al. 2024) |
| **Identification of safe harbors** | | | |
| Omics | Safe harbors | Key findings | Reference |
| transcriptome | Select T2 and T9 as candidate integration sites | Identification of regions with stable, high expression across 40 RNA-seq samples | (Pristovsek et al. 2019) |
| transcriptome  epigenome  TLA-seq | Identify genomic characteristics of favorable integration sites | Expression stability is partially governed by appropriate integration site selection | (Dhiman et al. 2020) |
| transcriptome  genome  epigenome | Approximately 11% genome exists as transcriptionally accessible regions | Narrow the candidate genomic regions for broadly applicable permissive loci | (Hilliard and Lee 2021) |
| transcriptome  genome  epigenome | Select 709 candidate stable integration sites | Three CRISPR/Cas9-targeted integration sites increased productivity, with sustaining stable productivity | (Hertel et al. 2022) |
| transcriptome  genome  epigenome | *Azin1*, *Sart1*, *Fosl2*, *Pde4a*, *Dop1b*, *Vgll4*, *Khdc4*, *Ddc* sites | Targeted integration at 8 candidate sites significantly enhanced transgene mRNA expression over *Fer1l4* | (Hilliard and Lee 2023) |
| genome | *IS14* site | Identification of transgene genomic sites generated by random integration | (Woo et al. 2024) |
| transcriptome  epigenome | Reporter expression from TRIP loci positively correlated with active chromatin marks | TRIP-identified transcriptional hotspots increased the mRNA levels up to 9.4-fold and fed-batch titers 5.6-fold, compared to a medium-expression control locus | (Tasan et al. 2025) |
| transcriptome  genome | 5 top-performing genomic hotspots were validated from 20 candidates | The identified genomic hotspots demonstrated a 2.2-fold to 15.0-fold enhancement in qₚ relative to *Fer1L4* and *Locus1* | (Lee et al. 2025) |

**Supplemental Table 2. Representative omics applications in functional gene engineering**

| **Identify novel regulators of apoptosis** | | | | |
| --- | --- | --- | --- | --- |
| Omics | Target genes | Key findings | Engineering | Reference |
| transcriptome proteome | *Pasg* (antiapoptotic) | Upregulation in butyrate-treated cells | Overexpression | (Yee et al. 2008) |
| transcriptome | *Tpt1* (antiapoptotic) | Downregulation at 33℃ | Overexpression | (Yee et al. 2009) |
| transcriptome | *Tp53inp1*  (proapoptotic) | Upregulation in butyrate-treated cells | Downregulation | (Birzele et al. 2010) |
| transcriptome | *Rassf5*  (proapoptotic) | Upregulation in response to hyperosmotic stress | Downregulation | (Shen et al. 2010) |
| exo-metabolome | *Ddah*  (antiapoptotic) | Dimethylarginine profile correlates with that of caspase. | Overexpression | (Chong et al. 2011) |
| proteome | *Tgm2*  *Clusterin*  (pro- and antiapoptotic) | Higher levels in the stationary phase | Downregulation  Overexpression | (Carlage et al. 2012) |
| transcriptome | *Anxa1*  *Lgals1*  (proapoptotic) | Up- and down-regulation in high producers | Downregulation | (Orellana et al. 2018) |
| proteome | *PPIB*  *LGMN*  (proapoptotic) | Upregulation in the apoptosis model | Downregulation | (Albrecht et al. 2018) |
| transcriptome | *TRAIL*  *BIM*  (proapoptotic) | Upregulation in the stationary phase | Downregulation | (Tossolini et al. 2018) |
| single‐cell transcriptome | *Hmxo1*  *Psap*  (proapoptotic) | Increase as cells advanced along the trajectory | Downregulation | (Tzani et al. 2021) |
| **Identify novel regulators of metabolism** | | | | |
| Omics | Target genes | Key findings | Engineering | Reference |
| transcritpome  proteome | *Acaa2* | Identification of differently expressed enzymes involved in fatty acid metabolism | Overexpression or downregulation | (Kang et al. 2014) |
| proteome  metabolome | *GGT1*  *GPX1*  *GSTM3*  *SOD1*  *SOD2* | Characterization of differently expressed proteins participating in GSH biosynthesis | Overexpression or downregulation | (Ali et al. 2019) |
| proteome | *GCLC*  *GCLM*  *HMGCS*  *MVD*  *FPPS* | Characterization of differently expressed proteins associated with GSH metabolism and cholesterol biosynthesis | Overexpression or downregulation | (Chevallier et al. 2020) |
| proteome | *PDHA1*  *DLAT*  *ME2* | Characterization of differently expressed proteins participating in pyruvate metabolism and TCA cycle | Overexpression | (Strasser et al. 2021) |
| proteome | IDH  d-LDHm  *GCLC*  *GCLM* | Identification of differently expressed proteins involved in TCA cycle and the elimination of  toxic metabolite methylglyoxa | Overexpression | (Kumar et al. 2022) |
| tanscriptome | *ENSCGRG000010029*  *GSS* | Correlate with qₚ | Overexpression  Downreglation | (Nelson et al. 2023) |
| **Identify regulators of glycosylation** | | | | |
| Omics | Target genes | Key findings | Engineering | Reference |
| proteome | *B4galt1*  *Fuca1*  *Man2a1*  *Man2b1* | Distinct expressed proteins correlate with differences in RTP quality (N-glycosylation) | Overexpression of B4galt1, Fuca1  Regulation of Man2a1/2b1 | (Park et al. 2017) |
| proteome | *FUT8*  *GMD* | Increase in mAb afucosylation coincided with a reduction in components of GDP-fucose synthesis and fucosylation pathway | Downregulation | (Nguyen Dang et al. 2019) |
| transcritpome  metabolome | *MAN1B1, MAN2A2, MAN2B1, MAN2B2, and MAN1A1*  *MGAT4B and MGAT1*  *B4GALT1* | Identification of differently expressed enzymes involved in N-glycosylation | Overexpression  or downregulation | (Sumit et al. 2019) |
| transcritpome  proteome  metabolome | *ALG13*  *MAN1A1*  *MAN2A1*  *MAN2A2* | Identification of differently expressed enzymes involved in N-glycosylation | Downregulation of *ALG13*  Overexpression of *Mannosidase* | (Lee et al. 2021) |
| **Identify novel regulators of secretory** | | | | |
| Omics | Target genes | Key findings | Engineering | Reference |
| transcritpome | *SRP9*  *JUN*  *PDIA3*  *HSPA8* | Identify SRP9, JUN, PDIA3, and HSPA8 as effective enhancers of RTPs | Overexpression | (Malm et al. 2022) |
| transcritpome | *Dpagt1* | Upregulation in tunicamycin-adapted cells | Overexpression | (Mishra and Mehra 2025) |
| transcritpome  proteome | *CUL4A*  *(Cullin 4A)*  *YWHAH*  *(14-3-3η)* | Identification of binding partners that promote sE1E2 secretion | Overexpression | (Wu et al. 2025) |

**Supplemental Table 3. Representative omics applications in vector optimization**

| **Promoter optimization** | | | |
| --- | --- | --- | --- |
| Omics | promoters | Key findings | Reference |
| transcriptome | endogenous S100a6 promoter | The activity of S100a6 is conditionally triggered at low temperatures | (Thaisuchat et al. 2011) |
| transcriptome | synthetic promoters using TF-TFRE pairs | Design and construct CHO promoters with precisely tunable activities | (Brown et al. 2017) |
| transcriptome  epignome | endogenous promoters of P1-D52, P2-CP, P3-actin, P8-HSP90 | The endogenous P8‐HSP90 exhibits promising stability compared to CMV P+E | (Nguyen et al. 2019) |
| transcriptome  genome | synthetic promoters with varying TFRE compositions | The top-performing synthetic promoter achieves transcriptional activities 2.5-fold and 8.3-fold higher than CMV and SV40 | (Johari et al. 2019) |
| transcriptome | endogenous *Actb*, *Ctsz*, *Hmox1*, *Hspa5*, and *Vim* promoters | These endogenous promoters show high gene transcription efficiency and consistent expression durability | (Tossolini et al. 2022) |
| **UTR engineering** | | | |
| Omics | UTRs | Key findings | Reference |
| transcriptome  translatome  (Ribo-seq) | synthetic 5’ UTRs by  mutating and evolving endogenous 5’ UTRs | Synthetic 5’UTRs boost protein yield and can combinatorially assemble to further enhance protein expression | (Cao et al. 2021) |
| transcriptome | 5 synthetic enhancers for the CMV promoter | Enhancer engineering of the CMV expression cassette boosts RTP yields | (Liu et al. 2024) |
| transciptome  miRNA transcriptome | miRNA targeted 3'UTRs fused downstream of DHFR | By using let-7f-targeted 3'UTRs to repress DHFR expression, it can improve RTP productivity in CHO cells by attenuating the selective markers | (Josse et al. 2018) |
| transcriptome | iPLUS in the 3'UTRs | iPLUS incorporation into the vector increases RTP productivity in CHO cells | (Reis-Claro et al. 2024) |
| **Signal peptide optimization** | | | |
| Omics | Signal peptide | Key findings | Reference |
| transcriptome | a panel of signal peptides | Novel vector designs enhance product titers by >1.8-fold in all cases | (O'Neill et al. 2023) |
| transcriptome | multiple novel signal peptides | Identify several novel signal peptides that boost HSA production | (Zhang et al. 2025b) |

**Supplemental Table 4. Representative omics applications in process optimization**

| **Media optimization** | | | |
| --- | --- | --- | --- |
| Omics | Media components | Key findings | Reference |
| transcriptome | Initial glucose  (30 mM, low 10 mM) | Low glucose concentration is not detrimental to CHO cells, supporting the design of glucose-limited fed-batch strategies for lactate management. | (Gowtham et al. 2017) |
| transcriptome, proteome, metabolome, lipidome | cysteine fed  (control, decreased by 15%, increased by 20%) | Cys restriction channels cellular resources into stress adaptation and anaplerotic energy salvage, effectively diverting them away from the biosynthetic pathways required for high RTP production | (Ali et al. 2020) |
| metabolome  proteome | fed system  (Cys or S-sulfocysteine (SSC)) | Compared to Cys control, SSC condition led to elevated levels of free cysteine and various thio-oxyanions, while reducing the abundance of cysteine trisulfide and thiocystine. | (Nguyen et al. 2023) |
| metabolome  transcriptome | without cysteine | CbCt cells suffer from metabolic depletion (reduced HCY/GSH) and ferroptosis in Cys-free conditions, while GnCbCt cells upregulate genes that support effective biosynthesis of cysteine. | (Greenfield et al. 2024) |
| proteome | Hydrolysate (soy, wheat, yeastolate) | Supplementation with soy, achieving the maximal μ, upregulates metabolic and proliferation-associated proteins, whereas supplementation with wheat, yielding the highest qₚ, alters chaperone (CRP55, PDI, and ERp57) levels. | (Kim et al. 2011a) |
| transcriptome | cottonseed hydrolysate | Supplementation with cottonseed enhanced cellular anabolic capacity and functional maturation, evidenced by the upregulation of Atf/homeobox factors, gluconeogenesis, and protein synthesis machinery, alongside the suppression of growth-restrictive Hippo signaling | (Kumar et al. 2021) |
| metabolome,  proteome | cottonseed hydrolysate | Cottonseed hydrolysate supplementation reprogrammed key cellular activities, including metabolism, transport, cell division, gene expression, and protein homeostasis. | (Dhara et al. 2023) |
| proteome | sodium butyrate | In 3 mM NaBu addition culture, the overexpression of Bcl-x(L) extends beyond merely inhibiting apoptosis to actively modulate diverse aspects of cell proliferation and survival. | (Blondeel et al. 2016) |
| proteome | sodium butyrate | After 25 hours post-butyrate treatment, 118 proteins exhibited significant abundance changes in CHO cells. | (Muller et al. 2017) |
| epigenome  transcriptome | sodium butyrate | Butyrate affects both well-established pathways (cell cycle, apoptosis) and those linked to hyperproductivity (energy metabolism, protein biosynthesis). | (Wippermann et al. 2017) |
| transcriptome | butyric acid | Butyric acid supplementation triggered simultaneous suppression of cell growth and remodeling of histone marks, which collectively enhanced downstream protein biosynthesis mechanisms. | (Schulze et al. 2022) |
| **Process optimization** | | | |
| Omics | Bioprocess parameters | Key findings | Reference |
| transcriptome proteome | Temperature  (37°C, 33°C) | Compared with 37°C control, the protein abundance of PDI, vimentin, NDK B, ERp57, and HSC70 was increased over two-fold at 33°C. | (Baik et al. 2006) |
| proteome | Temperature  (37°C, 31°C) | A temperature downshift triggered the specific differential expression of 23 proteins, functionally linked to key cellular processes. | (Kumar et al. 2008) |
| transcriptome | Temperature  (37°C, 31°C) | 31°C temperature shift recalibrated inducer effects on transcription and phenotype. | (Fomina-Yadlin et al. 2015) |
| phosphoproteome | Temperature  (37°C, 31°C) | Phosphoproteomic reprogramming induced by temperature downshift converges on key growth-regulatory processes and critical molecular functions. | (Henry et al. 2017) |
| transcriptome proteome  metabolome glycome | 3 defined pH (6.7, 6.9, and 7.1) | Differential pH setpoints modulate key intracellular pathways, thus shaping distinct profiles for qₚ, product titer, and quality. | (Lee et al. 2021) |
| transcriptome | Agitation rate [min-1]  (267, 700) | Distinct adaptation mechanisms are activated in response to different stresses. | (Sieck et al. 2014) |
| transcriptome | Agitation  (230 rpm, 280 rpm)  Volume  (5ml, 20 ml) | Low agitation combined with high volume suppressed cell growth and productivity, while high agitation mitigated the performance differences imposed by volume changes. | (Torres et al. 2021) |
| proteome  metabolome | Volume  (20 L, 5000 L) | In the production bioreactor, elevated copper concentration alleviates intermittent hypoxia, thereby reducing ROS accumulation and mitigating oxidative stress. | (Gao et al. 2016) |
| transcriptome  metabolome | Volume  (10 L, 15 mL) | The ambr 15 system serves as a valid miniaturized model for 10 L laboratory-scale systems. | (Alsayyari et al. 2018) |
| transcriptome  metabolome | Culture time  (from day 0 to day 5) | Establish an integrated, time-series analytical framework for CHO cell cultures | (Hsu et al. 2017) |
| transcriptome  metabolome  exo-metabolome | Culture time  (growth phase vs production phase) | Galactosylation and sialylation constitute kinetic limitations in glycosylation. | (Sumit et al. 2019) |
| transcriptome  (lncRNome) | Batch and fed-batch | lncRNAs with the most pronounced expression changes across culture phase or modes (batch vs. fed-batch) are potential targets regulating CHO cell growth | (Vito and Smales 2018) |
| exo-metabolome | Fed batch  (2 L) | The initial utilization of a genome-scale CHO model in an industrial fed-batch setting | (Calmels et al. 2019) |
| transcriptome | Fed batch  (ActiCHO and FortiCHO culture process) | Cell cycle arrest alongside activated mTOR signaling drives CHO cellular enlargement in response to nutrient availability. | (Pan et al. 2019) |
| exo-metabolome | Fed batch  (ranging from 10 L to 1000 L) | The metabolic patterns of CHO cells during fed-batch culture are consistent across different production scales. | (Vodopivec et al. 2019) |
| transcriptome  proteome | Fed batch  (normoxia and hypoxia) | Moderate hypoxia inhibits mitochondrial respiration and electron transport, while concurrently upregulating glycolysis and elevating ROS levels. | (Yin et al. 2025) |
